# Beyond Poisson’s Ratio: Material–Process Performance of Additively Manufactured Auxetic Lattices

**DOI:** 10.3390/ma19214506

**Published:** 2026-10-26

**Authors:** Barbara Schürger, Michal Hovanec, Peter Palička, Robert Szaniszló

**Affiliations:** 1 Department of Aviation Engineering, Faculty of Aviation, Technical University of Košice, Rampová 1731/7, 040 01 Košice, Slovakia; michal.hovanec@tuke.sk (M.H.); peter.palicka@tuke.sk (P.P.); 2 Department of Applied Mechanics and Mechanical Engineering, Faculty of Mechanical Engineering, Technical University of Košice, Letná 1/9, 042 00 Košice, Slovakia; robert.szaniszlo@tuke.sk

**Keywords:** auxetic metamaterials, re-entrant lattice, negative Poisson’s ratio, additive manufacturing, material extrusion, selective laser sintering, mechanical work, material selection

## Abstract

**Highlights:**

**What are the main findings?**

- One re-entrant lattice design was tested in nine material/process variants, with 10 specimens per variant (*N* = 90).
- Maximum force, displacement at peak load, structural stiffness and mechanical work depended strongly on the variant (Kruskal–Wallis ε^2^ = 0.908–0.942), with coefficients of variation below 5% within each variant.
- PLA reached the highest mean maximum force, whereas PETG accumulated the highest mean mechanical work up to 14 mm (7.9% more than PLA; 95% simultaneous confidence interval 1.2–14.5%) despite a 4.3% lower mean maximum force.

**What are the implications of the main findings?**

- Statistical inference was based on the replicated mechanical metrics; optical values of the effective Poisson’s ratio are reported as descriptive observations.
- PA12 produced by selective laser sintering reached a higher maximum force and mechanical work than PA12 produced by fused deposition modeling; this contrast characterizes the two tested material–process combinations rather than the manufacturing route alone.
- Material selection for auxetic lattices therefore requires force, displacement and work criteria in addition to Poisson’s ratio.

**Abstract:**

Auxetic lattices are usually tailored through geometry, whereas the effect of the constituent material and manufacturing route at fixed architecture has received little replicated experimental attention. One re-entrant lattice design was produced in nine material/process variants and tested in quasi-static tension (10 specimens per variant, *N* = 90): polylactic acid (PLA), impact-modified PLA, glycol-modified polyethylene terephthalate (PETG), acrylonitrile butadiene styrene (ABS), acrylonitrile styrene acrylate (ASA), UltraT and Flex 3D by fused deposition modeling (FDM), and polyamide 12 (PA12) by FDM and by selective laser sintering (SLS). Maximum force F_max_, crosshead displacement at peak load u(F_max_), archived structural stiffness k and mechanical work W_14_ up to 14 mm displacement depended strongly on the variant (*p* < 0.001; Kruskal–Wallis ε^2^ = 0.908–0.942). PLA reached the highest mean F_max_ (68.48 ± 2.40 N), whereas PETG accumulated the highest mean W_14_ (622.80 ± 28.03 N mm), 7.9% more than PLA (95% simultaneous confidence interval 1.2–14.5%), despite a 4.3% lower F_max_. SLS PA12 exceeded FDM PA12 by 11.0% in F_max_ and by 21.9% in W_14_. The W_14_ ranking refers to the 14 mm limit, and k is interpreted as an archived descriptor. Previously processed optical measurements gave effective Poisson’s ratios of −1.043 to −1.052 for eight variants; these values are reported descriptively, and a linear finite-element model illustrates the auxetic mechanism. Force, displacement at peak load and mechanical work should therefore complement Poisson’s ratio in material selection for auxetic lattices.

## 1. Introduction

Mechanical metamaterials derive their effective macroscopic response from their internal architecture as well as from the properties of the parent material. Auxetic metamaterials have a negative effective Poisson’s ratio, i.e., they expand transversely under axial tension. Re-entrant lattices are among the most established auxetic architectures because their global response follows directly from the bending and rotation of inclined ligaments, which links unit-cell geometry to structural kinematics [1,2,3,4,5].

Additive manufacturing (AM) makes such geometries available in polymers, composites and metals, but a manufactured lattice is a coupled architecture–material–process system. Even at unchanged nominal cell geometry, deposition direction, interlayer bonding, variation in ligament thickness, porosity, thermal history and surface quality can change the stiffness, force capacity, damage initiation and force–displacement response [3,6,7,8]. Poisson’s ratio alone therefore does not describe engineering performance; peak load, deformation, stiffness and the mechanical work up to a prescribed displacement must also be considered.

Previous material comparisons support this view but have usually covered fewer materials or varied the geometry at the same time. Vyavahare and Kumar compared re-entrant structures made from acrylonitrile butadiene styrene (ABS) and polylactic acid (PLA) by fused deposition modeling (FDM) under compression and reported material-dependent stiffness and specific energy absorption while also varying the cell geometry [9]; their later work examined the same materials under flexural loading using regression and machine-learning methods [10]. Dong et al. showed that printing parameters strongly affect the peak force and energy-related response of 3D-printed re-entrant structures [7], and a subsequent study of PLA, ABS, nylon and multi-material designs found that the Poisson response of single-material lattices is governed mainly by geometry, whereas peak force, stiffness and energy absorption depend strongly on the material [11].

Another line of research improves energy-related performance or programs effective properties by modifying the architecture itself. Modified, reinforced and graded re-entrant designs have substantially increased the absorbed energy or specific energy absorption under compression [12,13], and recent reviews identify geometry optimization, manufacturing strategy and multifunctional design as major research themes [8,14]. Effective elastic properties can also be controlled through topology. Spinodal-decomposition-inspired morphologies generated from the dimensionless Cahn–Hilliard equation allow the homogenized anisotropic elastic constants to be mapped to morphology parameters and inverted for target properties [15]; hierarchical combinations of heterogeneous and homogeneous cell arrangements program the elastic modulus and Poisson’s ratio over wide positive and negative ranges [16]; and multifunctional or hybrid lattices offer further routes to a programmable Poisson response [17,18]. These approaches vary the architecture to reach target properties. The present study addresses the complementary question: how much does the mechanical performance change when the re-entrant architecture is held constant and the material/process variant is the experimental variable?

The manufacturing route is part of this question. Studies of polyamide 12 (PA12) produced by selective laser sintering (SLS) show that tensile properties, failure strain and Poisson response depend on build orientation and wall thickness [19,20,21]. Because the two PA12 groups of the present study differ in polymer grade, density, porosity, orientation and thermal history as well as in the manufacturing route, their comparison is treated as a replicated material–process contrast within a common lattice design.

The distinction between auxetic kinematics and structural performance matters in lightweight and aerospace design, where mechanical metamaterials have been proposed for morphing and adaptive structures, lightweight cellular cores and impact-oriented concepts [22,23,24,25], and where additive manufacturing enables topology-optimized and functionally integrated components [26,27,28]. Such applications require a balance of deformation mode, stiffness, force capacity, mass, durability and energy-related response, which Poisson’s ratio alone cannot capture.

The experimental approach can also support broader materials-development frameworks that combine laboratory testing, simulation and data-driven material selection. Experiments at fixed architecture yield material/process-response descriptors that can be used in predictive or artificial intelligence (AI)-assisted screening without mixing geometric and material effects.

This work differs from the authors’ earlier study in Materials, which numerically compared re-entrant, chiral and anti-chiral architectures with four metallic material models and two loading directions [29]. Here, topology and loading are fixed, and the focus is on replicated experimental benchmarking of nine polymer material/process variants. The study combines one re-entrant geometry tested with 10 specimens per variant (*N* = 90); a statistical analysis of maximum force, displacement at peak load, structural stiffness and mechanical work; descriptive optical observations of the effective Poisson’s ratio together with an illustration of the auxetic mechanism by a linear finite-element method (FEM) model; a replicated comparison of PA12 produced by FDM and SLS; and a multi-metric performance map.

The comparison between glycol-modified polyethylene terephthalate (PETG) and PLA is of particular interest because the two materials rank differently in different metrics: PETG had the higher mean mechanical work and archived stiffness, whereas PLA reached the higher maximum force. Because the stiffness metric describes the whole lattice specimen rather than the Young’s modulus of the polymer, this contrast is reported as an observed structural ranking of the tested variants, and literature on polymer morphology, interlayer cohesion and process-induced anisotropy in material extrusion [30,31,32,33,34,35,36] provides the context for its discussion.

**Figure 1:**
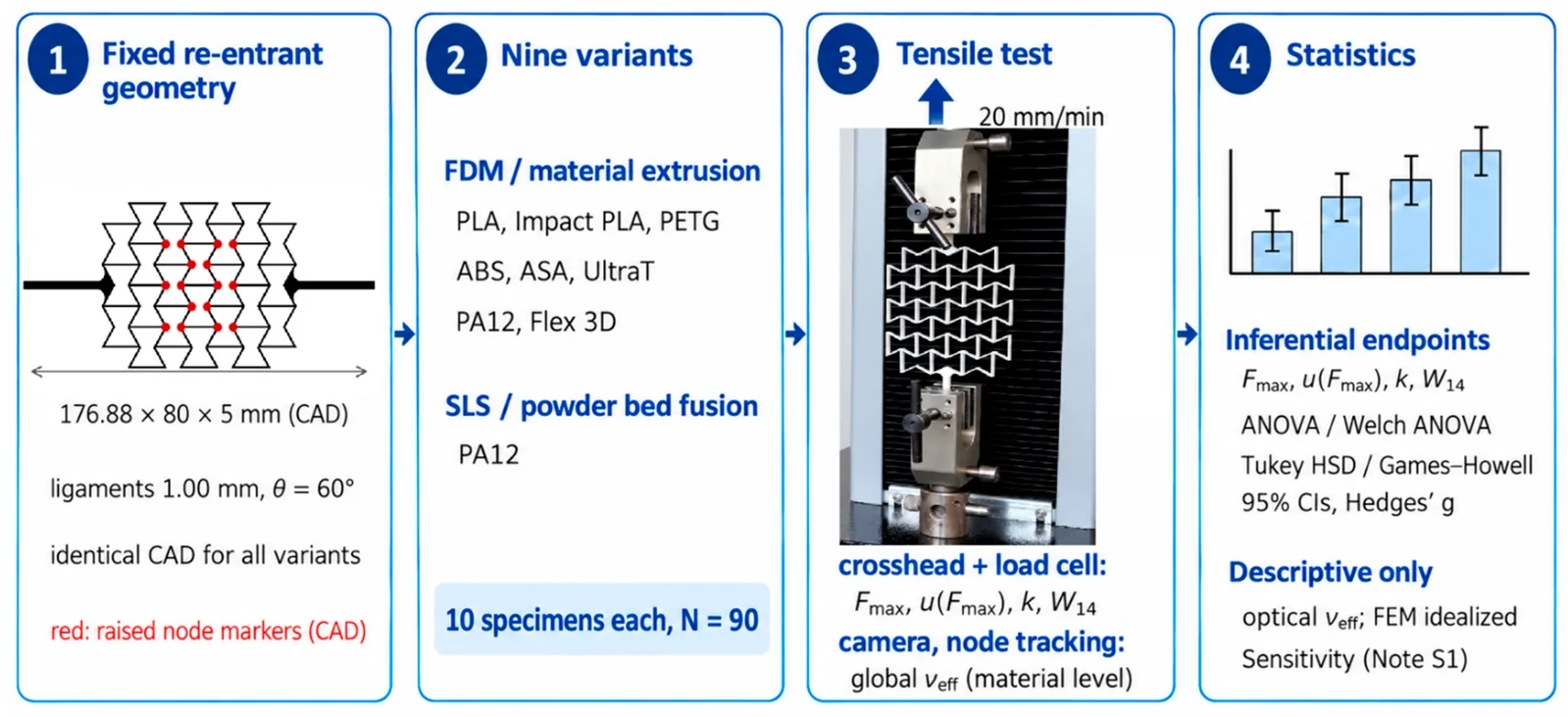
Study design: (1) re-entrant specimen drawn from its computer-aided design (CAD) geometry (red: raised node markers); (2) nine material/process variants with 10 specimens each (N = 90); (3) quasi-static tensile test with crosshead-based metrics and optical marker tracking; (4) statistical evaluation.

The objective is therefore to separate response measures that are mainly governed by the architecture from those that depend strongly on the material/process variant. We hypothesized that the fixed re-entrant cell would produce a similar global auxetic response across the materials, whereas the replicated force, displacement and work metrics would differ substantially. Only the mechanical metrics are tested statistically; the optical Poisson’s ratio values serve as descriptive observations. The overall experimental workflow, including specimen geometry, material/process variants, tensile testing and statistical evaluation, is summarized in Figure 1.

## 2. Materials and Methods

### 2.1. Specimen Geometry

**Figure 2:**
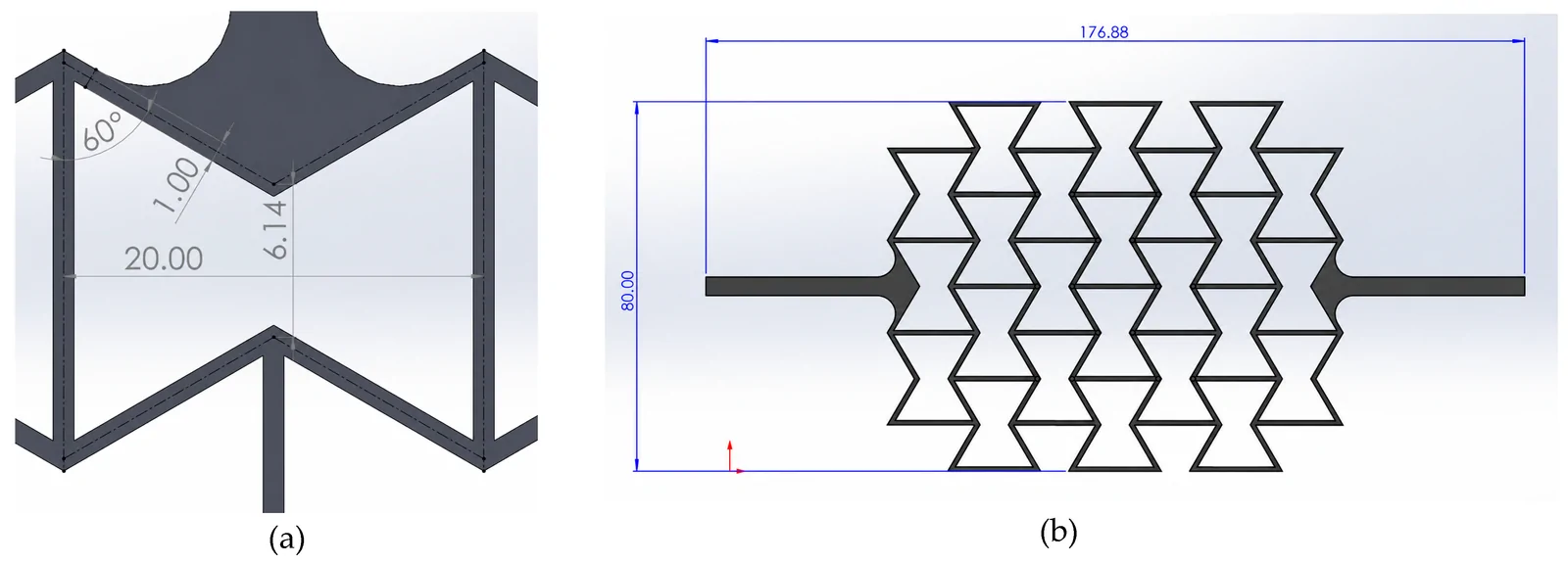
Geometry of the re-entrant specimen: (**a**) enlarged detail of the unit-cell geometry showing the parameters θ, t, h and w; (**b**) overall geometry and dimensions of the tensile specimen. All dimensions are nominal CAD dimensions in mm. The arrows indicate the orientation of the CAD coordinate system.

All material groups used one nominal re-entrant lattice design and were tested under the same boundary conditions. The computer-aided design (CAD) geometry, verified from the mesh stored in the original slicer projects, measures 176.88 × 80.00 mm in plan and 5.00 mm in thickness; 16 raised node markers (diameter approximately 1 mm, height 0.75 mm) increase the local thickness to 5.75 mm (Figure 2). The lattice occupies approximately 98 mm of the specimen length and is connected by fillets to 4.0 mm wide end arms used for gripping. All lattice ligaments are 1.00 mm wide, except for the two lateral boundary members, which are 2.0 mm wide. The unit cell is defined by the angle θ = 60° between the inclined ligaments and the cell walls, the ligament width t = 1.00 mm, the distance h = 6.14 mm between opposite re-entrant vertices and the cell width w = 20.00 mm (centerline dimensions). These are nominal CAD dimensions. Inspection of earlier FDM specimens of the same design revealed local thickening and thinning of nominally identical ligaments, material accumulation from travel moves and distortion of edge cells [37]. The dimensions of the tested specimens were not measured, so differences between groups are interpreted as combined effects of material, processing and manufactured geometry.

The re-entrant cell deforms by coupled bending and rotation of its inward-inclined ligaments. Under axial tension, the cells open laterally, which produces a positive transverse strain and hence a negative effective Poisson’s ratio. Geometry optimization is outside the scope of this article; the architecture serves as a common kinematic platform for comparing the mechanical response of the material/process variants.

### 2.2. Additive Manufacturing

The CAD geometry was prepared in SolidWorks 2024 (Dassault Systèmes, Vélizy-Villacoublay, France) and sliced in Bambu Studio for a Bambu Lab A1 printer (Bambu Lab, Shenzhen, China) with a 0.4 mm nozzle. Eight of the nine groups were produced by material extrusion (MEX), referred to here as FDM; the ninth group was produced by SLS (see below). According to the documentation of the original study, the specimens were produced with identical 3D-printing parameters [37]. The slicing configuration of the design is preserved in two Bambu Studio projects, which differ only in the color assigned to the node markers (black or white); the white-marker variant was used for the black PETG and Flex 3D specimens. Both files were re-saved on 9 October 2026 with Bambu Studio 02.07.01 and contain the geometry and the process settings. They therefore document the slicing configuration of the design; the settings of the individual print jobs, including material-specific temperatures, were not recorded separately.

Table 1 summarizes the common FDM configuration and indicates the source of each entry: settings saved in the projects (P), filament-profile defaults stored in the projects (D) and information confirmed by the documentation of the original campaign (C). The saved process profile is the standard “0.20 mm Standard @BBL A1” system profile with a 0.20 mm layer height, two wall loops with the classic wall generator, 15% grid sparse infill, five top and three bottom solid layers and no supports. The specimens were printed flat, so the 5 mm thickness comprises 25 layers and the ligament walls are formed by contour roads that follow the ligament direction in each layer. With a 0.42 mm outer-wall line width, a 1.00 mm ligament holds one outer-wall road from each adjacent cell contour (approximately 0.84 mm together), which leaves less than one line width. The ligaments are therefore expected to consist of these two roads with possible gap fill, whereas inner walls and sparse infill fit only where the width exceeds approximately 1.7 mm, i.e., in the 2.0 mm boundary members, the fillets and the 4.0 mm end arms. This road layout is derived from the saved settings and the CAD geometry; it was not examined on sectioned specimens.

Table 2 summarizes the manufacturing route and material designation of all nine variants. The projects contain the Bambu Lab PLA Basic, ABS and PETG Basic system filament profiles. Their default temperatures are listed in Table 2 for reference only; they are stored slicer defaults, not verified printing temperatures of the specimens. The extrusion and bed temperatures, cooling and effective speeds actually used for each group and the moisture conditioning of the hygroscopic PA12 filament [38,39] were not recorded. The study therefore compares material/process variants produced from one nominal architecture with the common slicing configuration of Table 1, not with individually optimized process windows, and the results characterize the specimens as manufactured.

The loading direction was aligned with the dominant extrusion direction so that the axial load is not carried directly across the weaker interlayer interfaces. FDM anisotropy and process variability remain, however: bead geometry, local fusion, ligament thickness, material accumulation, stringing and edge distortion can affect the local bending stiffness and damage initiation, particularly in the slender ligaments and nodes that control the re-entrant deformation.

Ten PA12 specimens were produced by SLS and compared with the ten PA12 FDM specimens. Because this comparison includes the SLS process conditions, powder and post-processing as well as the manufacturing route itself, it is reported as a replicated material–process contrast, and the SLS group is characterized by its measured response.

### 2.3. Material/Process Variants

Nine material/process variants were evaluated: PLA, impact-modified PLA (Impact PLA), PETG, ABS, acrylonitrile styrene acrylate (ASA), UltraT, PA12 (FDM), PA12 (SLS) and Flex 3D; Impact PLA, UltraT and Flex 3D are commercial designations. The set covers comparatively stiff thermoplastics, toughened and engineering polymers, a highly compliant filament and a powder-bed-fused PA12 group (Table 2). All comparisons rely on the measured structural response of the manufactured specimens rather than on nominal parent-material properties, and the conclusions therefore refer to the tested material/process variants rather than to the corresponding polymer families in general.

**Figure 3:**
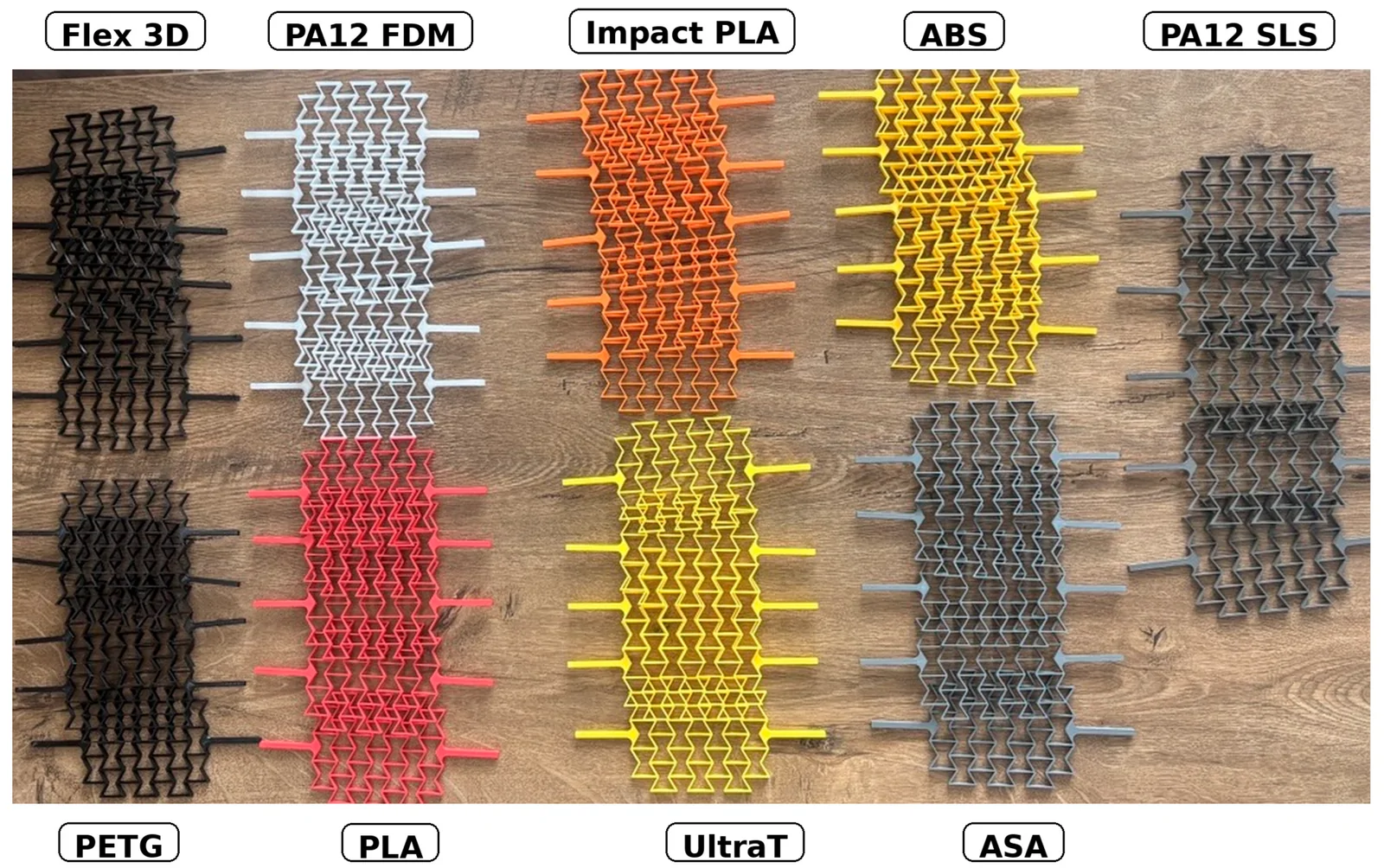
Representative re-entrant specimens of the nine material/process variants (five of the ten tested specimens per variant are shown).

Figure 3 shows representative specimens of all nine variants. For a compact presentation, five specimens per variant are displayed; ten specimens per variant were tested (N = 90).

### 2.4. Tensile Testing and Image Acquisition

Quasi-static tensile tests were performed on a KASON WDW-10 electromechanical universal testing machine (Jinan Kason Testing Equipment Co., Ltd., Jinan, China) at a constant crosshead speed of 20 mm/min. The machine frame is rated at 10 kN; force was measured with a CHCONTECH CH-SD-500kg load cell (rated capacity 500 kg ≈ 4.90 kN; limit load 750 kg ≈ 7.35 kN), so the individual peak forces (24.8–72.0 N) correspond to 0.5–1.5% of the rated capacity. The specimens were clamped by their end arms in the standard grips, with the specimen axis aligned with the loading axis [37]. The machine export contained time, force, crosshead displacement and machine-calculated stress, and all displacement-based metrics therefore use the crosshead displacement u [37]. Ten specimens were tested for each of the nine variants (90 tests) under indoor laboratory conditions.

Images were acquired with the Dantec Dynamics Q-450 system (Dantec Dynamics A/S, Skovlunde, Denmark), operated in the Istra4D software environment (Dantec Dynamics A/S, Skovlunde, Denmark), using SpeedSense 9070 high-speed cameras with complementary metal–oxide–semiconductor (CMOS) sensors (nominal maximum resolution 1280 × 800 pixels) [40,41]. The cameras were positioned perpendicular to the specimen plane, the specimen was illuminated by a Dedolight DLH400D daylight source (400 W; approximately 5600 K, Dedo Weigert Film GmbH, Munich, Germany).and the system was calibrated with a reference length placed in the specimen plane; the calibration sensitivity and accuracy of the same platform have been examined previously [40]. Force and crosshead displacement were used for the mechanical metrics, whereas the longitudinal and transverse dimensional changes needed for the effective Poisson’s ratio were obtained from the images (Section 2.5), because the crosshead displacement includes machine and grip compliance and contains no transverse information.

**Figure 4:**
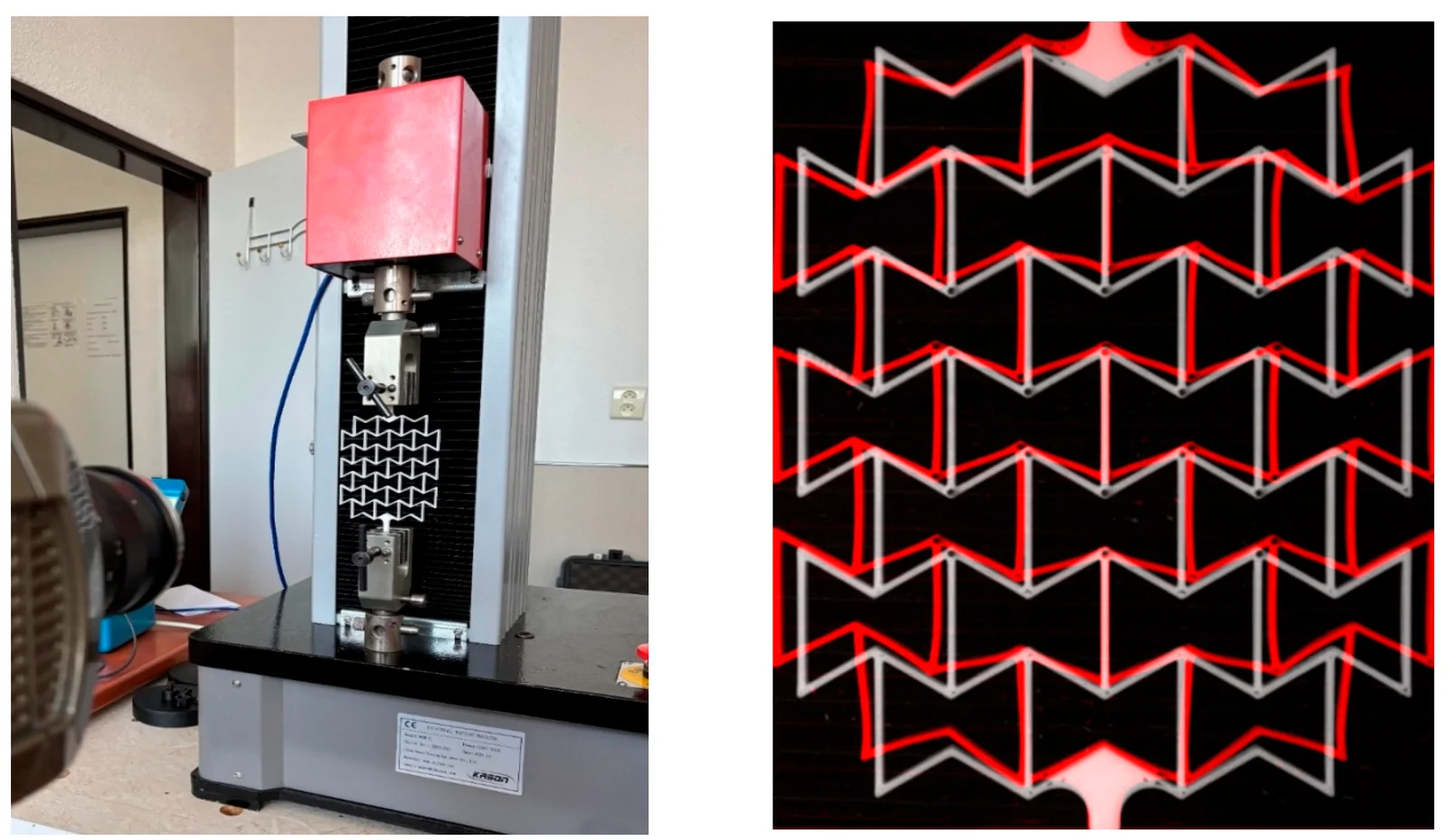
Test arrangement: KASON WDW-10 universal testing machine, Dantec Dynamics Q-450 camera system with two SpeedSense 9070 cameras, and Dedolight DLH400D daylight illumination.

The experimental arrangement, including the tensile testing machine, imaging system and representative optical tracking of the auxetic specimen, is shown in Figure 4.

All groups were tested with the same load cell and set-up, so a gain or offset error of the load cell would shift all groups equally and largely cancel in between-group comparisons. Manufacturer accuracy data for the 25–72 N range, a calibration certificate for the test period and records of the preload and zeroing procedures are not part of the available documentation; the force values are therefore interpreted comparatively within this test series. The 2.96 N difference in mean maximum force F_max_ between PLA and PETG (0.06% of the rated capacity) is statistically resolved against the within-group scatter (95% simultaneous confidence interval 0.49–5.44 N), and its absolute magnitude should be interpreted with this in mind.

The ten specimens per variant provide specimen-level replication of the four mechanical metrics defined in Section 2.7, which are the variables analyzed statistically; the complete dataset of 90 specimens is given in Supplementary Table S1. The optical effective Poisson’s ratio is available as one value per variant and is reported descriptively.

### 2.5. Optical Evaluation of Effective Poisson’s Ratio

Documented procedure. The Q-450 system was used only for image acquisition; no digital image correlation (DIC) was performed. The effective Poisson’s ratio ν_eff_ was obtained by tracking lattice nodes and manually applied markers in the image plane. According to the documentation of the original study [37], the images were exported in Hierarchical Data Format version 5 (HDF5) and processed with a custom Python 3.10 workflow: grayscale conversion, filtering and contrast enhancement, threshold-based segmentation, detection of nodes and markers with the OpenCV library, tracking of the detected positions through the image sequence, and calculation of the longitudinal and transverse dimensions of the tracked region. A You Only Look Once (YOLO) object-detection model (Ultralytics) served only as a detection aid when the classical identification of a node failed; it did not define any dimension or strain.

The global effective Poisson’s ratio of the tracked region is the negative ratio of its transverse engineering strain ε_trans_ to its axial engineering strain ε_ax_:

(1) $$\nu_{eff}\;=\;-\frac{\varepsilon_{trans}}{\varepsilon_{ax}}\;=\;-\frac{\left(\frac{\Delta W}{W_{0}}\right)}{\left(\frac{\Delta L}{L_{0}}\right)}$$

where L_0_ and W_0_ are the initial axial and transverse dimensions of the tracked region and ΔL and ΔW are their changes during loading. A negative ν_eff_ indicates transverse expansion under axial tension. ν_eff_ is an effective strain ratio of the anisotropic lattice structure, not the Poisson’s ratio ν of the parent material; values slightly below −1 are therefore physically admissible [1,2,5].

Available results and accuracy. One previously processed ν_eff_ value is available for each of eight variants as presented in Section 3.1. The PETG and Flex 3D specimens were black, whereas the marker detection used in [37] was designed for dark markers on light specimen surfaces; ref. [37] therefore reports no optical values for these two materials. The corresponding values in Table 3 were obtained in a later evaluation of specimens printed with white node markers (the white-marker variant of the design, Section 2.2); because this evaluation is not described in [37], the two values are interpreted with corresponding caution. The Python script originated from a related diploma thesis and is no longer available. The evaluation settings (reference region L_0_ and W_0_, evaluation frame, number of image sequences per value, and handling of tracking losses and out-of-plane motion) are therefore known only as described above, and no accuracy value can be assigned to the individual results. The sensitivity of the global ratio to the gauge region is illustrated by local point-pair evaluations on a separate PLA specimen with MATLAB R2022b (MathWorks, Natick, MA, USA) and X-Sight tracking, which gave ν_eff_ between −0.48 and −1.23 depending on the marker pair [37]. These values are therefore used as descriptive material-level observations, separately from the replicated mechanical endpoints and from the FEM results.

### 2.6. Idealized Finite-Element Model

An idealized finite-element model was used to illustrate the deformation mechanism of the re-entrant architecture independently of the optical measurements [37]. The model was solved in ANSYS Workbench 2022 R2 for the nominal specimen geometry (lattice of approximately 98.4 × 80 mm with end arms). The end face of the lower arm was fixed, and a tensile force was applied to the end face of the upper arm along the specimen axis. The analysis was linear-elastic and geometrically linear, formulated as a planar problem and discretized with quadratic planar elements. PLA was modeled as isotropic with Young’s modulus E = 2939 MPa and Poisson’s ratio ν = 0.33 (ANSYS Workbench material library). In a mesh-convergence study with characteristic element sizes of 0.50, 0.40, 0.25 and 0.20 mm, ν_eff_ converged from −1.017 to −1.026, −1.032 and −1.033; a 0.25 mm mesh with local refinement at the ligament junctions (approximately 329,285 nodes and 191,644 elements) was adopted.

The numerical ν_eff_ was evaluated with Equation (1), where ΔW is the range (maximum minus minimum) of the displacement field in the transverse X direction and ΔL is the range in the axial Y direction, normalized by W_0_ = 80 mm and L_0_ = 176.88 mm. At a force of 30 N, ΔW = 1.443 mm and ΔL = 3.093 mm gave ν_eff_ = −1.032. A load sweep from 10 to 50 N in 5 N steps returned the same value at every level, as expected for a linear analysis, and the maximum von Mises stress at 30 N (45.3 MPa) remained below the library yield strength of PLA (56.9 MPa). With the same geometry, mesh, supports and load, an orthotropic PLA model representing FDM anisotropy, with Young’s moduli E_x_ = 2669, E_y_ = 2583 and E_z_ = 2208 MPa, Poisson’s ratios ν_xy_ = 0.43 and ν_yz_ = ν_zx_ = 0.37, and shear moduli G_xy_ = 919 and G_yz_ = G_zx_ = 844 MPa in the principal material directions x, y and z, changed ν_eff_ from −1.032 to −1.033 [37]. Values obtained with the material models of the other polymers (−1.031 to −1.035) are listed in Supplementary Table S8. Further inputs, such as the element type designation, the plane-stress or plane-strain option with its thickness and the Poisson’s ratios of the other material models, are not part of the model documentation and are therefore not reported. Because the numerical ratio depends on the chosen reference dimensions and the model is linear and idealized, the FEM results and the optical observations are presented separately as complementary illustrations.

### 2.7. Mechanical Metrics

Four metrics were determined for each tested specimen: the maximum force F_max_ (highest recorded force), the crosshead displacement at peak load u(F_max_), the structural stiffness k and the mechanical work W_14_ defined in Equation (2). All four were calculated during the original campaign from the machine-recorded force–displacement data and are used here as archived values (Table S1). k is the slope of a linear fit to the approximately linear rising part of the force–displacement response; its fitting interval is documented only for a reference PLA evaluation (2–8 mm crosshead displacement, k = 7.09 N/mm) [37]. Because the time series of the 90 tests are not available for re-analysis, k cannot be recalculated with a uniform window. Supplementary Note S1 examines the influence of the fitting window using the single-specimen curves documented in [37] (one curve per variant, digitized in Figure S1), which share the specimen design and test procedure with the 90 tests but whose relationship to the specimens of Table S1 is not known. These curves show an initial seating region up to approximately 1.5–2 mm, which a window starting at 2 mm excludes. A common 2–8 mm window reproduced the archived k of the PLA and ABS curves within 0.2% and deviated by −20.7% to +19.9% for the other variants; for PA12 (FDM) and PA12 (SLS), the archived values exceeded the steepest regression slope over any 3 mm window. Within a single curve, k varied by 7–28% across windows between 1 and 9 mm, and the PETG–PLA difference ranged from 1% to 23% (Table S6, Figure S2a). These results indicate that the archived k values were not obtained with one fixed window, and differences of about 20% lie within this window sensitivity. k is accordingly referred to as the archived structural stiffness, a descriptor of the complete specimen–grip system rather than a standardized stiffness or a Young’s modulus, and the mechanistic interpretation and the performance map (Section 3.7) are based on F_max_ and W_14_.

(2) $$W_{14}=\int_{0}^{14\;mm}F(u)\;du$$

In Equation (2), F(u) is the force at crosshead displacement u. W_14_ is the mechanical work done on the specimen up to a common crosshead displacement of 14 mm; it allows specimens with different force–displacement responses to be compared under the same imposed displacement. When F_max_ occurred before 14 mm, the post-peak response up to 14 mm was included. The W_14_ values were computed in the original campaign and are used as archived values.

The 14 mm limit was chosen after the force–displacement records had been obtained, as a displacement reached in all records, so that every specimen contributes over the same interval [37]. Its position relative to the peak load differs between groups (Table S1). All specimens of Impact PLA, PETG, ABS and ASA reached F_max_ before 14 mm, so their W_14_ includes post-peak response; all specimens of PA12 (FDM) and Flex 3D reached F_max_ at or beyond 14 mm, so their W_14_ covers only the pre-peak response; and PLA (3 of 10 specimens), UltraT (4 of 10) and PA12 (SLS) (5 of 10) lie on both sides of the limit. On the digitized single-specimen curves, the post-peak part contributed 31% of W_14_ for PETG and 49% for ASA. Rankings based on W_14_ therefore apply to the selected displacement. On the same curves, PETG had the largest work at every limit between 6 and 16 mm, but its advantage over PLA decreased from 23% at 6 mm to 7% at 14 mm and 3% at 16 mm, and the order of ABS, ASA and PA12 (SLS) changed above 14 mm (Figure S2b). Trapezoidal integration of the digitized curves reproduced their archived W_14_ values within 0.6%, which is consistent with a standard area integration.

(3) $$W_{rel}=\left(\frac{W_{i}}{W_{max}}\right)\times 100\%$$

In Equation (3), W_i_ is the mean W_14_ of variant i and W_max_ is the highest group mean, obtained for PETG; the relative mechanical work W_rel_ therefore expresses each group mean as a percentage of the PETG mean. Because the tests were quasi-static and the displacement was measured at the crosshead, W_14_ is a comparative structural work metric and not a measure of material toughness, impact energy, specific energy absorption or crashworthiness.

The force–displacement time series of the 90 tests are stored on the testing-machine computer at the authors’ former workplace and were not accessible for re-analysis; the statistical analysis is therefore based on the four specimen-level metrics in Table S1, and the constraint-based curve visualization originally submitted as Supplementary Figure S1 was withdrawn. Supplementary Note S1 shows that these metrics admit a range of curve shapes: for the group means, curves sharing F_max_, u(F_max_) and k admit W_14_ values between 47% and 110% of the measured W_14_, and the force at 6 mm can differ by 8–15 N even when W_14_ is also prescribed (Table S7). The results are therefore presented as metric-based comparisons.

### 2.8. Statistical Analysis

For each mechanical metric and group (n = 10), the mean, standard deviation (SD) and coefficient of variation (CV = SD/mean) were calculated. Normality was screened with the Shapiro–Wilk test (*p* < 0.05 in 1 of 36 group–metric combinations: k of Impact PLA, *p* = 0.033), and homogeneity of variances was assessed with the median-centered Levene test. F_max_ met the equal-variance criterion (Levene *p* = 0.147) and was analyzed by one-way analysis of variance (ANOVA) with Tukey’s honestly significant difference (HSD) test. u(F_max_), k and W_14_ showed unequal variances (Levene *p* = 0.038, 0.020 and 0.011) and were analyzed by Welch’s ANOVA with Games–Howell pairwise comparisons; ANOVA results are reported as F(df1, df2), the test statistic with its numerator and denominator degrees of freedom. As a distribution-free sensitivity analysis, tie-corrected Kruskal–Wallis tests were performed with the effect size ε^2^ = (H − m + 1)/(N − m), where H is the Kruskal–Wallis statistic, m = 9 is the number of groups and N = 90 is the total number of specimens. For the principal pairwise contrasts, mean differences are reported with 95% simultaneous confidence intervals (CIs) from the same post hoc procedures, together with the standardized effect size Hedges’ g.The significance level was α = 0.05, and *p* denotes the multiplicity-adjusted *p*-value for pairwise comparisons. A non-significant pairwise result is reported as “no statistically significant difference was detected” and is not taken as evidence of equivalence. All omnibus and pairwise results (Supplementary Tables S2–S5) were recomputed from the specimen-level values in Table S1 for this revision. Specimens printed in the same job may share process conditions; the *p*-values and CIs therefore describe the tested specimen series and may understate process-level variability.

## 3. Results

### 3.1. Effective Poisson’s Ratio

**Figure 5:**
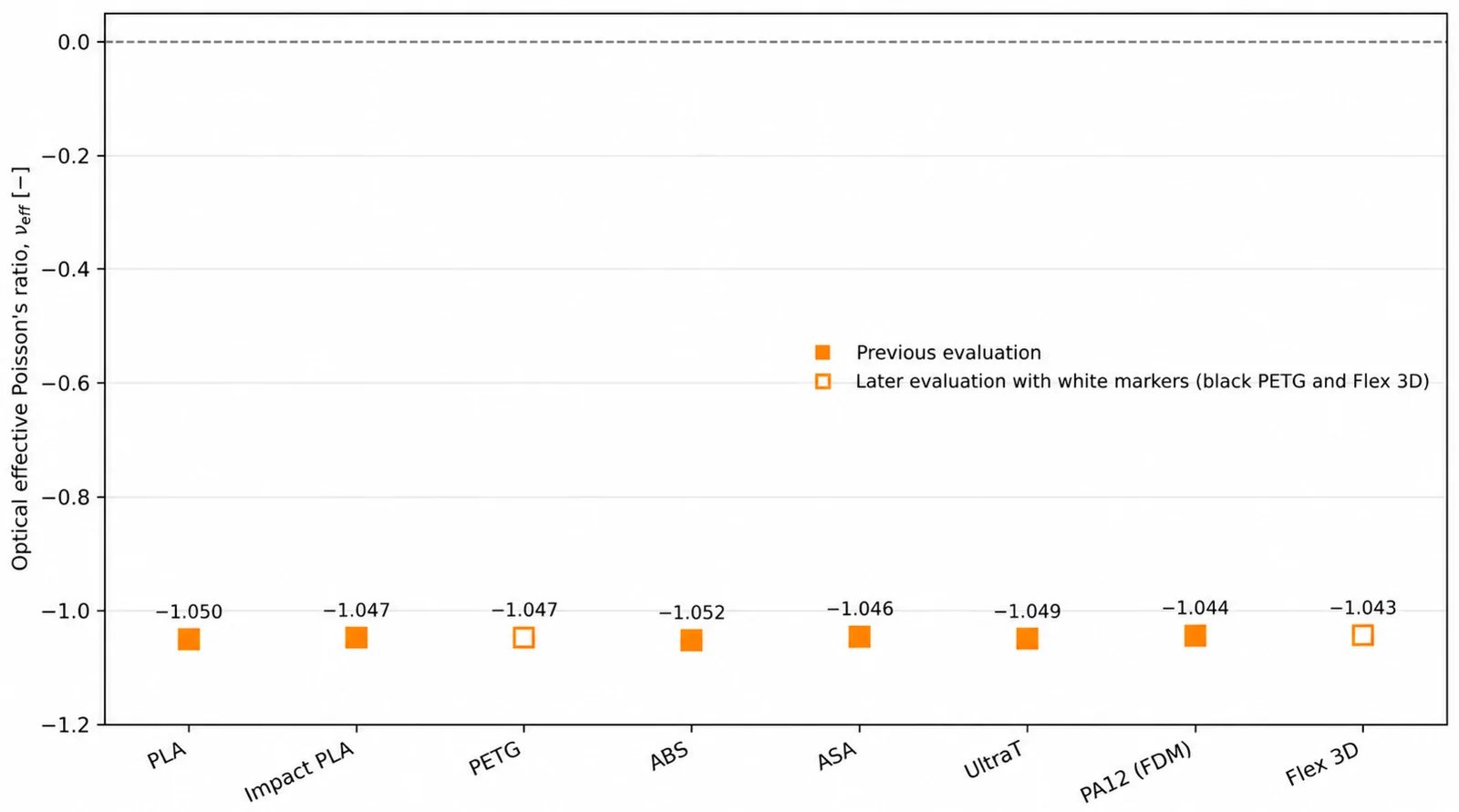
Global effective Poisson’s ratio νeff obtained from optical marker tracking for the eight evaluated material variants. Filled symbols represent previously reported measurements [37], while open symbols indicate the later evaluation of PETG and Flex 3D specimens using white node markers. The dashed line indicates νeff = 0.

Table 3 lists the previously processed optical ν_eff_ values, while Figure 5 provides their graphical comparison across the eight evaluated material variants. All eight evaluated variants showed a negative ν_eff_ between −1.043 and −1.052, although their mean F_max_ ranged from 26 to 68 N. As described in Section 2.5, these values are descriptive: the narrow range indicates a consistently auxetic response of the evaluated variants, whereas the material dependence of ν_eff_ remains a question for dedicated, replicated strain measurements. The idealized FEM model reproduces the auxetic mechanism through rotation and bending of the inclined ligaments; its values are listed separately in Table S8.

In the idealized FEM model, replacing isotropic PLA by the orthotropic representation changed ν_eff_ only from −1.032 to −1.033, the total deformation from 3.093 to 3.088 mm and the maximum equivalent stress from 45.3 to 45.6 MPa [37]. In this model, the global kinematics are therefore governed by the cell geometry rather than by the elastic constants; testing this quantitatively for the printed specimens requires replicated strain measurements.

### 3.2. Force–Displacement Response and Maximum Force

All statistical tests, effect sizes, confidence intervals, pairwise comparisons and rankings in Section 3.2, Section 3.3, Section 3.4, Section 3.5, Section 3.6, Section 3.7 and Section 3.8 are based on the 90 specimen-level values in Supplementary Table S1; values are given as mean ± SD.

**Figure 6:**
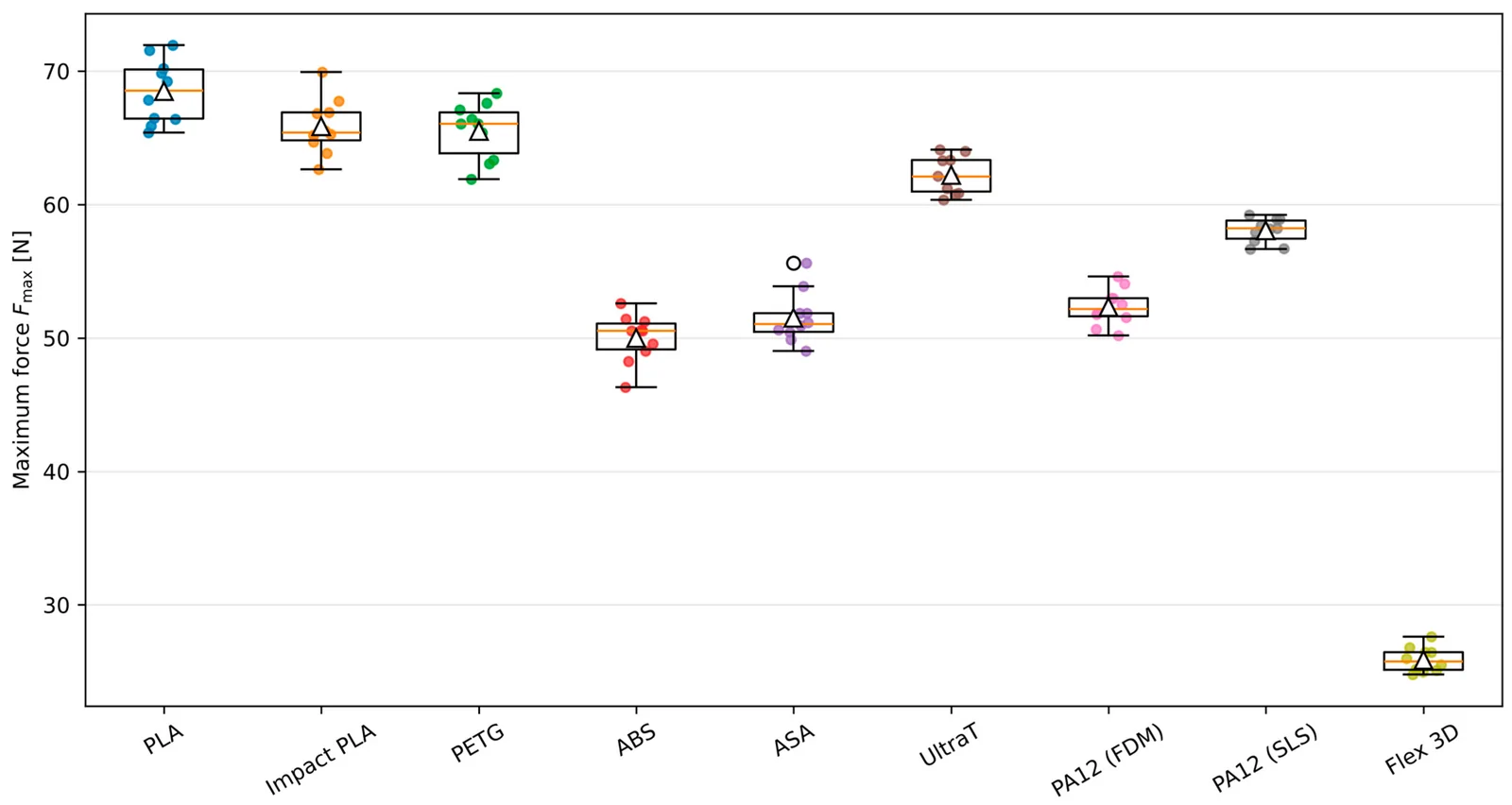
Maximum force F_max_ of the 10 tested specimens per material/process variant. Boxes show the median and interquartile range, whiskers extend to 1.5 times the interquartile range, triangles mark the group means and points show the individual specimens.

PLA had the highest mean F_max_ (68.48 ± 2.40 N), followed by Impact PLA (65.86 ± 2.07 N), PETG (65.52 ± 2.11 N) and UltraT (62.21 ± 1.41 N); Flex 3D had the lowest (25.89 ± 0.93 N) (Figure 6). The effect of the material/process variant on F_max_ was significant (one-way ANOVA, F(8, 81) = 564.25, *p* < 0.001). In Tukey’s HSD test, PLA exceeded PETG by 2.96 N (95% simultaneous CI 0.49–5.44 N; *p* = 0.008) and Impact PLA by 2.62 N (0.15–5.10 N; *p* = 0.029), whereas no statistically significant difference was detected between Impact PLA and PETG (*p* = 1.000). These two differences amount to approximately 4% of the group means, and their lower confidence limits are close to zero; they should be read together with the force-measurement considerations in Section 2.4. PA12 (SLS) also exceeded PA12 (FDM) in F_max_ (58.05 ± 0.91 vs. 52.31 ± 1.40 N; difference 5.74 N, 95% CI 3.26–8.21 N; *p* < 0.001).

### 3.3. Displacement at Peak Load and Structural Stiffness

**Figure 7:**
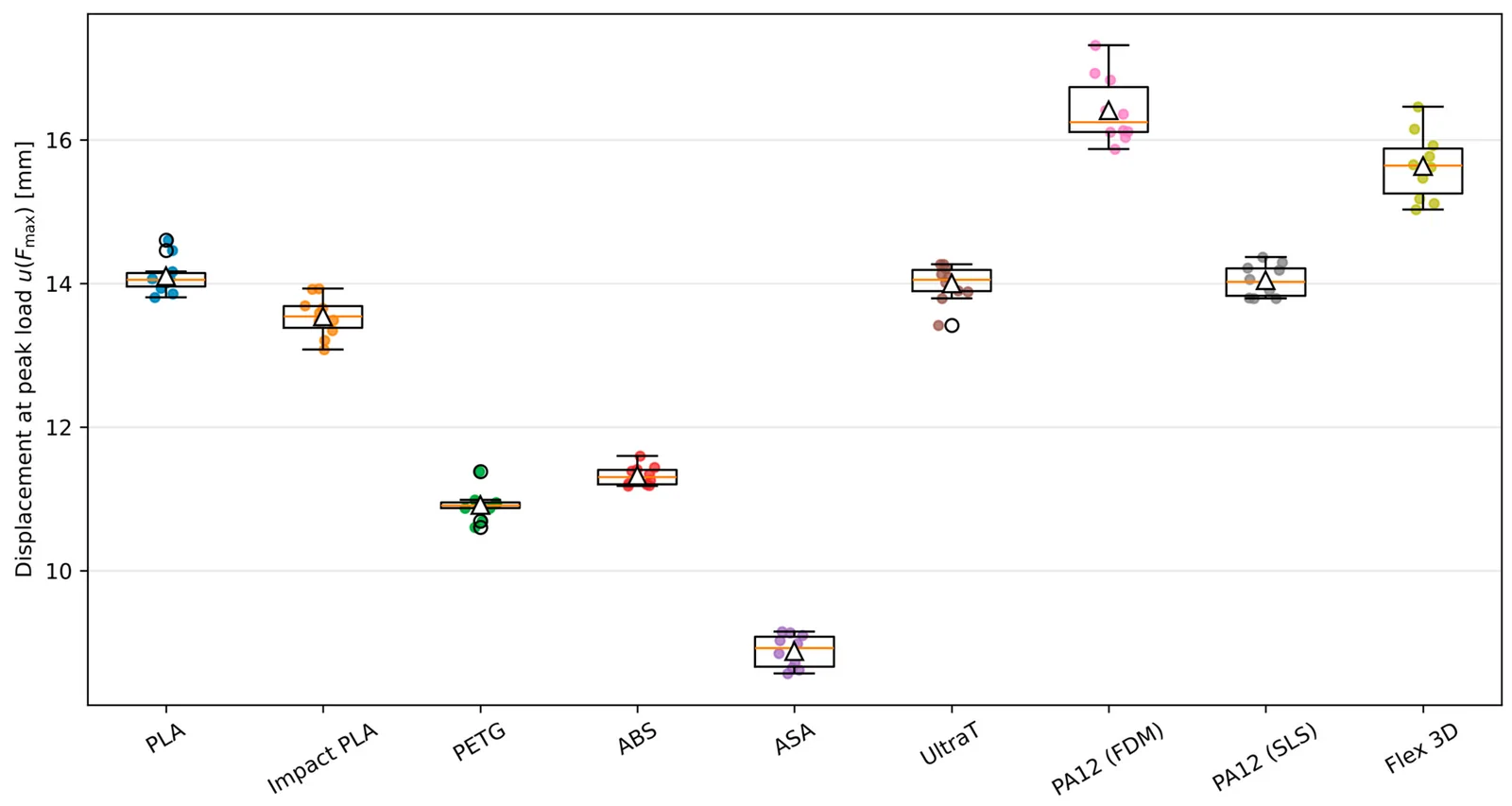
Crosshead displacement at peak load u(F_max_) of the 10 tested specimens per variant (box plot as in Figure 6).

The displacement at peak load and the archived stiffness ranked the variants differently. PA12 (FDM) had the largest mean u(F_max_) (16.41 ± 0.47 mm), followed by Flex 3D (15.64 ± 0.46 mm), whereas ASA reached F_max_ at the smallest mean displacement (8.88 ± 0.23 mm) (Figure 7). The effect on u(F_max_) was significant (Welch’s ANOVA, F(8, 33.48) = 687.32, *p* < 0.001). PA12 (FDM) reached F_max_ at a larger displacement than PA12 (SLS) (16.41 ± 0.47 vs. 14.04 ± 0.22 mm; Games–Howell *p* < 0.001). u(F_max_) describes the position of the peak load and is used in this sense rather than as a measure of deformation capacity.

**Figure 8:**
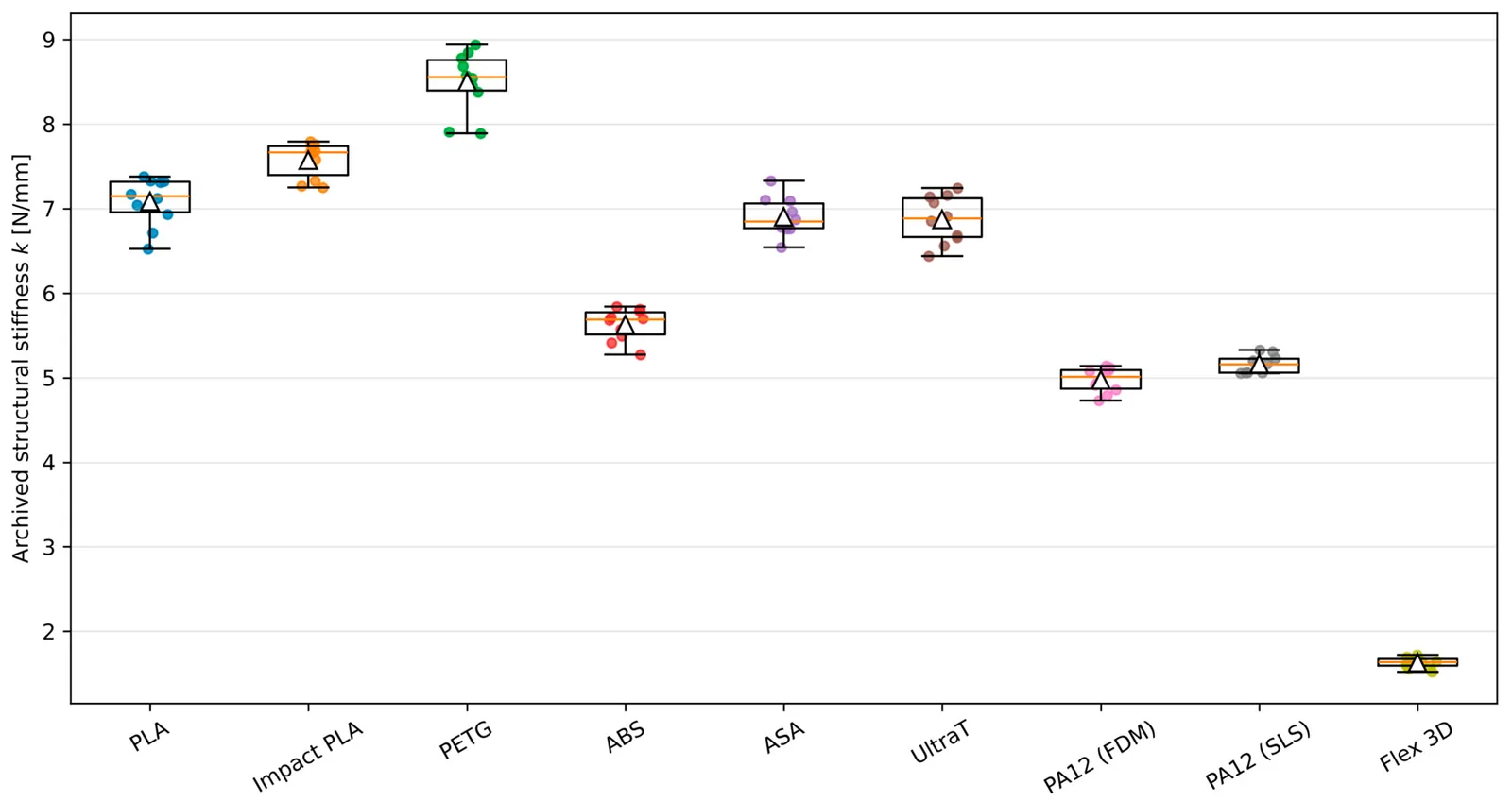
Archived structural stiffness k of the 10 tested specimens per variant (box plot as in Figure 6). The archived values were not obtained with one fixed fitting window (Section 2.7).

PETG had the highest mean archived stiffness (8.50 ± 0.36 N/mm), followed by Impact PLA (7.58 ± 0.21 N/mm) and PLA (7.08 ± 0.29 N/mm); Flex 3D had the lowest (1.63 ± 0.06 N/mm) (Figure 8). The effect was significant (Welch’s ANOVA, F(8, 32.80) = 2686.34, *p* < 0.001), and Games–Howell comparisons showed a higher mean k for PETG than for PLA (difference 1.42 N/mm, 95% simultaneous CI 0.90–1.93 N/mm; *p* < 0.001) and for Impact PLA (*p* < 0.001). Because k varied by up to 28% with the fitting window on the single-specimen curves (Section 2.7), these are differences in the archived values. No statistically significant difference in k was detected between PA12 (SLS) and PA12 (FDM) (5.16 ± 0.11 vs. 4.97 ± 0.15 N/mm; 95% CI −0.02 to 0.39 N/mm; *p* = 0.090). On the single-specimen curves, however, k extracted with a common window differed between these two variants by 15–27% for every window, so this result is not taken as evidence of similar stiffness.

### 3.4. Mechanical Work up to 14 mm Displacement

**Figure 9:**
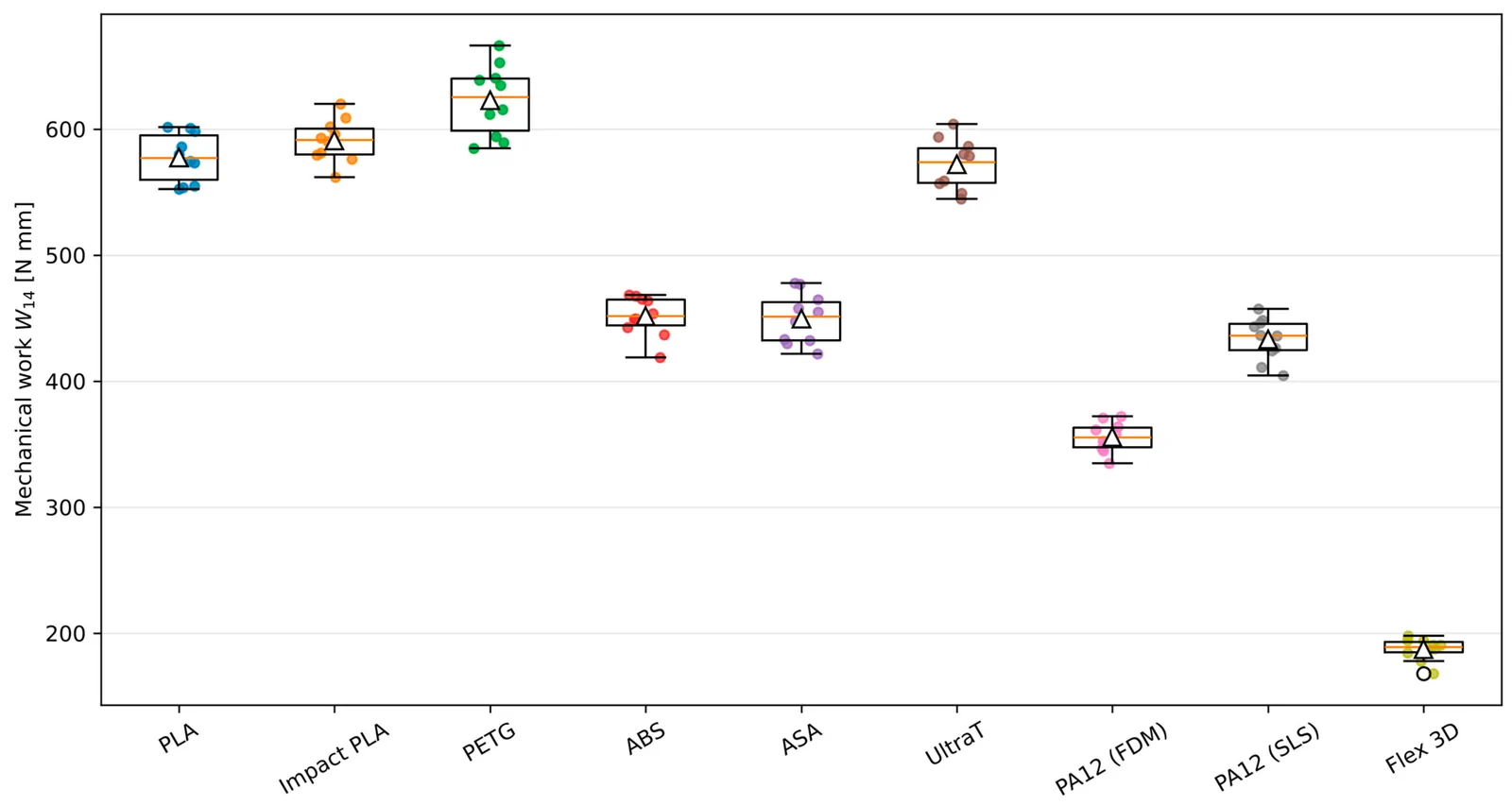
Mechanical work W_14_ up to 14 mm crosshead displacement of the 10 tested specimens per variant (box plot as in Figure 6). W_14_ depends on the 14 mm limit (Section 2.7).

PETG had the highest mean W_14_ (622.80 ± 28.03 N mm) and serves as the reference for W_rel_ (100%). It was followed by Impact PLA (590.78 ± 16.96 N mm), PLA (577.46 ± 19.29 N mm) and UltraT (572.12 ± 19.77 N mm), whereas Flex 3D had the lowest mean W_14_ (187.18 ± 8.79 N mm); the highest group mean was thus 3.33 times the lowest (Table 4, Figure 9). The effect on W_14_ was significant (Welch’s ANOVA, F(8, 33.45) = 1049.21, *p* < 0.001). PETG exceeded PLA by 45.3 N mm, or 7.9% (95% simultaneous CI 7.0–83.6 N mm, i.e., 1.2–14.5% of the PLA mean; Games–Howell *p* = 0.014), despite a 4.3% lower mean F_max_; the lower confidence limit is close to zero, so the size of this difference is imprecise. No statistically significant difference was detected between PETG and Impact PLA (*p* = 0.121; 95% CI −5.3 to 69.3 N mm) or between Impact PLA and PLA (*p* = 0.773). All PETG specimens reached F_max_ at 10.61–11.38 mm, so their W_14_ includes approximately 3 mm of post-peak response, whereas 7 of 10 PLA specimens reached F_max_ at or beyond 14 mm. The PETG–PLA ranking in W_14_ therefore applies to the 14 mm limit; on the single-specimen curves, the same order held for limits between 6 and 16 mm, with a margin that decreased as the limit increased (Figure S2b). F_max_ and W_14_ therefore provide related but different rankings.

### 3.5. Comparison of PA12 Produced by FDM and SLS

**Figure 10:**
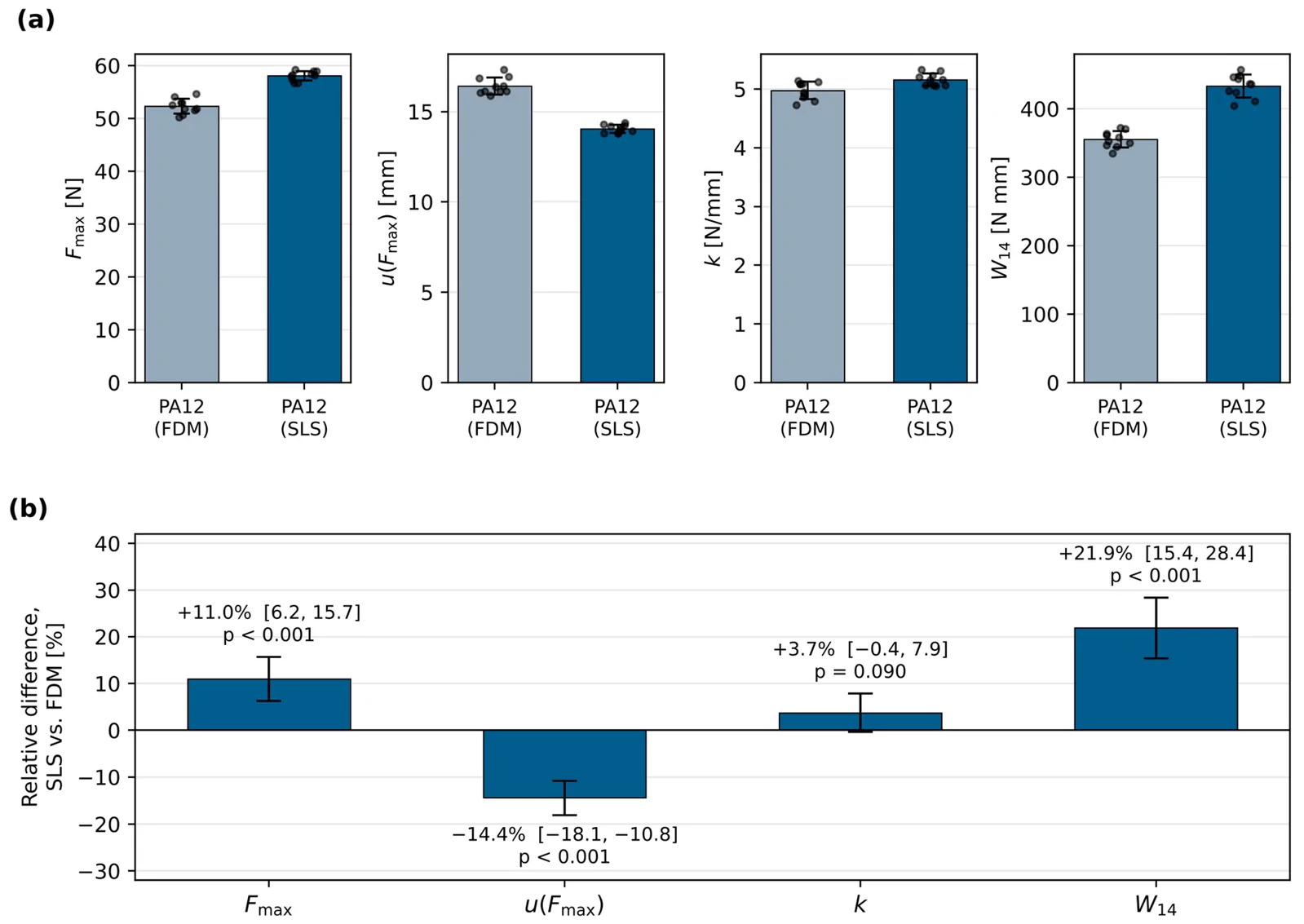
Comparison of PA12 (SLS) with PA12 (FDM). (**a**) Group means of F_max_, u(F_max_), k and W_14_ (bars) with ±1 SD (error bars) and individual specimens (points), n = 10 per variant. (**b**) Relative difference in the SLS mean from the FDM mean (bars) with 95% simultaneous confidence intervals (error bars; Tukey’s HSD for F_max_, Games–Howell for the other metrics) and adjusted *p*-values. The confidence intervals in (**b**) describe the uncertainty of the difference between the means, not the scatter of individual specimens.

The two PA12 variants differed in a metric-dependent way (Figure 10). Relative to PA12 (FDM), PA12 (SLS) had an 11.0% higher mean F_max_ (58.05 ± 0.91 vs. 52.31 ± 1.40 N; difference 5.74 N, 95% CI 3.26–8.21 N; Tukey *p* < 0.001), a 21.9% higher mean W_14_ (433.26 ± 16.76 vs. 355.50 ± 11.99 N mm; difference 77.8 N mm, 95% CI 54.6–100.9 N mm; Games–Howell *p* < 0.001) and a 14.4% lower mean u(F_max_) (14.04 ± 0.22 vs. 16.41 ± 0.47 mm; 95% CI −2.97 to −1.77 mm; *p* < 0.001). The 3.7% difference in mean k was not statistically significant (*p* = 0.090) and lies within the window sensitivity of k (Section 3.3). Five of the ten PA12 (SLS) specimens, but none of the PA12 (FDM) specimens, reached F_max_ before 14 mm, so part of the W_14_ difference reflects the different position of the 14 mm limit relative to the peak load. These results characterize the two tested PA12 variants, which differ in grade-, density- and process-related factors as well as in the manufacturing route (Section 4.4).

### 3.6. Post-Peak Response

The analysis of the 90 tests is based on scalar metrics, and the test program did not include failure-mode classification or fractography; the shape of the loading curve, the post-peak load reduction, damage initiation and the fracture modes of the individual variants are therefore outside the scope of this study. Post-peak information for these specimens comes indirectly from the position of u(F_max_) relative to the 14 mm limit, which indicates whether W_14_ includes post-peak response (Section 2.7). The single-specimen curves in Figure S1 show abrupt load drops for some variants (e.g., ASA and Flex 3D) and gradual post-peak softening for others; with one curve per variant, these observations are illustrative.

### 3.7. Multi-Metric Material Selection

**Figure 11:**
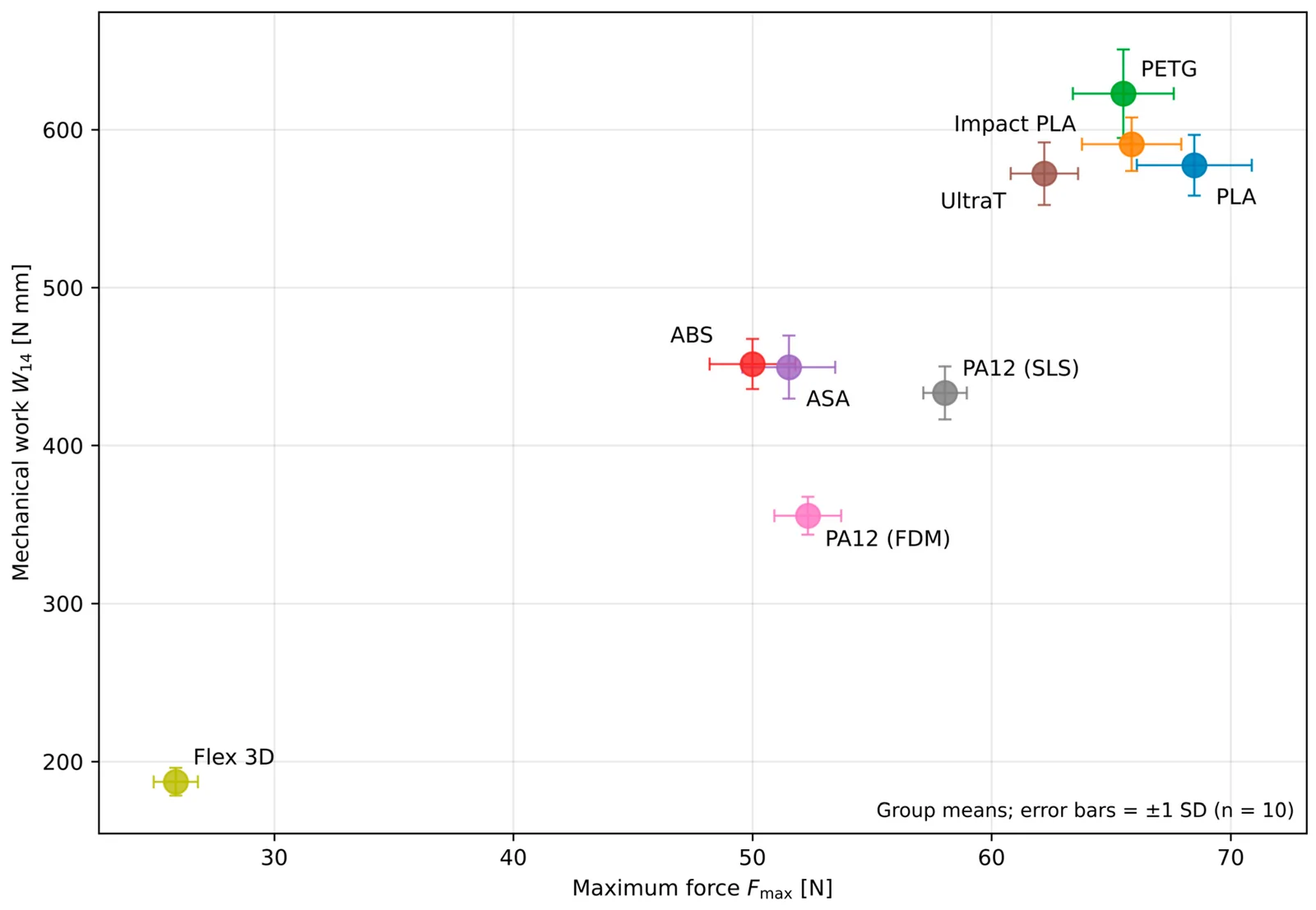
Performance map of the group means of F_max_ and W_14_ (n = 10 per variant; error bars ±1 SD). W_14_ refers to the 14 mm limit. The map shows trade-offs without weighting coefficients; k is omitted because its archived values depend on the fitting window (Section 2.7).

The performance map (Figure 11) shows metric-dependent trade-offs rather than a single ranking. PETG and Impact PLA occupy the region of highest W_14_ at a mean F_max_ slightly below that of PLA; PLA provides the highest mean peak load; and UltraT reaches a W_14_ close to that of PLA at a lower F_max_. ABS and ASA occupy an intermediate region; the PA12 variants combine intermediate F_max_ with lower W_14_, and Flex 3D combines the lowest F_max_ and W_14_ with a comparatively large u(F_max_). The appropriate material/process variant therefore depends on the required balance of peak load, displacement at peak load and mechanical work; the map does not impose a composite score.

### 3.8. Statistical Significance and Experimental Variability

All four mechanical metrics showed significant group effects in the primary omnibus tests (Table 5). Within-group scatter was low: the CV ranged from 1.56% to 3.77% for F_max_, from 1.22% to 2.95% for u(F_max_), from 2.04% to 4.24% for k and from 2.87% to 4.70% for W_14_. The Kruskal–Wallis tests also gave *p* < 0.001 for all four metrics, with ε^2^ = 0.924 for F_max_, 0.929 for u(F_max_), 0.942 for k and 0.908 for W_14_. The differences between the variants are therefore large relative to the within-group scatter of this campaign, which characterizes specimen-level rather than batch-to-batch or machine-to-machine variability. Adjusted pairwise comparisons for all 36 pairs of variants are given in Supplementary Tables S2–S5.

Table 6 summarizes the principal pairwise contrasts discussed below. The large contrasts, such as the PA12 SLS–FDM differences in F_max_ and W_14_, are estimated precisely, whereas the PLA–PETG and PLA–Impact PLA contrasts in F_max_ and the PETG–PLA contrast in W_14_ have lower confidence limits close to zero, so their magnitude is uncertain.

## 4. Discussion

### 4.1. Auxetic Kinematics and Material/Process-Dependent Mechanical Response

The two types of evidence in this study differ in strength. The mechanical metrics differed strongly between the variants across 90 tests, with very large effect sizes. The optical ν_eff_ values were negative for all eight evaluated variants and fell within a narrow range, and the idealized FEM model reproduces the auxetic mechanism of the architecture. As single, descriptive observations, the optical values show that all evaluated variants responded auxetically; whether ν_eff_ is independent of the material is a question for replicated strain measurements.

This distinction agrees with the geometric interpretation of re-entrant lattices [1,2,3,4,5] and with comparative studies showing that material properties, printing direction and material distribution modify the load-bearing and energy-related response [6,7,9,10,11]. At fixed nominal architecture, the force–displacement–work response differed substantially among the tested variants; these differences combine material, process and manufactured-geometry effects and apply to quasi-static tension. The fixed-architecture benchmark complements approaches that program effective properties by changing the topology, such as spinodal-inspired [15] or hierarchical [16] architectures: those approaches vary the geometry for a given base material, whereas the present data quantify the spread produced by material/process selection at fixed nominal geometry. Determining how far architecture and material effects can be separated would require both kinds of variation, together with a validated strain measurement, within one study.

### 4.2. Performance Trade-Offs

Replication shows the material ranking as a set of trade-offs. Mean F_max_ ranges from 25.89 to 68.48 N, mean k from 1.63 to 8.50 N/mm and mean W_14_ from 187.18 to 622.80 N mm, while all CV values remain below 5%. The very large ε^2^ values show that the differences between the variants are large relative to the scatter within them. The best-performing variants are, however, not ranked identically by every metric.

PLA had the highest mean F_max_, whereas PETG had the highest mean W_14_ and k. No statistically significant difference was detected between Impact PLA and PETG in F_max_ or W_14_; this does not show that the two variants are equivalent (Table 6). The data therefore identify no single best material but support selection according to the required combination of peak load, displacement at peak load and mechanical work at a prescribed displacement.

The PETG–PLA contrast (higher mean F_max_ for PLA, higher mean W_14_ for PETG) is reported as a structural observation; polymer morphology, crystallinity, porosity, interlayer bond strength, melt rheology and fracture surfaces, which would be needed to identify its mechanism, were outside the scope of the test program. The literature indicates plausible contributors. In material extrusion, the strength of inter-road and interlayer bonds depends on contact, neck growth and chain interdiffusion within a material-specific time–temperature window [30,33,36]; for PLA, interfacial healing and crystallinity jointly determine the tensile strength [30,34]; process parameters measurably change the response of both PETG and PLA [31,35]; and published tensile data report higher strength and modulus for PLA but a larger ultimate strain for PETG [32]. The last observation is consistent with the present ranking, which refers to the 14 mm limit, and is offered as context. Likewise, the higher mean k of PETG does not imply a higher Young’s modulus of the polymer: k describes the whole specimen, including ligament bending and rotation, road geometry, local imperfections and machine and grip compliance. For Impact PLA, UltraT and Flex 3D, which are characterized by their commercial designations, the responses are treated as observed properties of the tested variants without assigning a toughening or molecular mechanism [42].

### 4.3. Information Added by the Mechanical Work W_14_

W_14_ adds information that F_max_ alone cannot provide, because it integrates the force over a common displacement interval. PETG had a 4.3% lower mean F_max_ than PLA but a 7.9% higher mean W_14_; this difference was statistically significant, but its 95% simultaneous CI (1.2–14.5% of the PLA mean) shows that its size is imprecise. The difference follows from the timing of the peak load: PETG reached F_max_ at about 11 mm with a steeper rising response, whereas most PLA specimens were still approaching their peak at 14 mm, so post-peak response contributes to W_14_ only for PETG (on the single-specimen curves, PETG retained 92% of its peak load at 14 mm). A higher peak force therefore does not imply more work at a prescribed displacement.

The common 14 mm limit makes the specimens comparable by imposing the same global displacement on each of them and by retaining post-peak response when F_max_ occurs earlier. The resulting ranking, however, depends on the limit. On the single-specimen curves, PETG led at every limit between 6 and 16 mm, but its advantage over PLA decreased from 23% to 3% as the limit increased (Figure S2b), and the order of the intermediate variants changed above 14 mm. For a component designed for a known stroke, the work should therefore be evaluated at that stroke. As noted in Section 2.7, W_14_ is a comparative structural work metric derived from quasi-static crosshead displacement, and no impact or crashworthiness claim is made from the present data.

### 4.4. PA12 Produced by FDM and SLS

Within the tested PA12 variants, the SLS specimens had a significantly higher mean F_max_ and W_14_ and a lower u(F_max_) than the FDM specimens (Table 6, Figure 10). The largest relative difference occurred in W_14_ (21.9%), partly because the 14 mm limit lies at a different position relative to the peak load in the two variants. The difference in k was not statistically significant and lies within the window sensitivity of k, which reached 15–27% between the corresponding single-specimen curves. The two PA12 variants thus produced different performance envelopes with the same nominal architecture.

The comparison reflects the manufacturing route together with polymer grade, relative density, porosity, surface state, build orientation, powder history and thermal history. Moisture content is a further relevant factor for PA12, as studies of PA12 and other polyamides in material extrusion emphasize moisture control and pre-drying [38,39], and SLS PA12 studies show that build direction, wall thickness and process history affect the tensile properties [19,20,21]. The result is therefore a replicated contrast between the two specific PA12 variants tested here rather than a general ranking of SLS and FDM.

### 4.5. Measurement and Reproducibility Considerations

The low CV values show good repeatability among the tested specimens; reproducibility across manufacturing batches, printers, operators or laboratories would require a dedicated multi-batch study, and the experimental unit here is the individual specimen. FDM-specific variation in bead geometry, interlayer fusion, ligament thickness and thermal distortion can affect the local response even at fixed nominal geometry [6,7,8,14,37]. The optical ν_eff_ values are methodologically separate from the mechanical dataset; as single, previously processed values per variant, they are used descriptively, and inferential statements concern the mechanical metrics. Section 5 and summarize the data basis of the study and the resulting scope of interpretation.

### 4.6. Engineering Relevance

The engineering value of the study lies in material screening rather than component qualification. Morphing and adaptive aerospace structures require a prescribed deformation mode together with adequate stiffness and load transfer, and auxetic cellular cores are being investigated for lightweight and impact-oriented sandwich concepts [22,23,24,25]; additive manufacturing makes such architectures feasible in forms that are difficult to produce conventionally [26,27,28]. The present benchmark shows that the same auxetic architecture can be combined with very different force–displacement–work responses. A compliant morphing skin or fairing that must expand transversely at a low actuation force, for example, would favor low-force variants such as Flex 3D or PA12 (FDM), whereas an insert or core loaded to a prescribed stroke would favor variants with high work at that stroke, such as PETG and Impact PLA. These uses are prospective and would require the additional evidence listed below.

These results are consistent with a two-stage design proposition. In the first stage, a cellular architecture is selected to obtain the required auxetic kinematics; in the present study, all evaluated variants showed a negative ν_eff_, in line with the mechanism illustrated by the idealized FEM model, while the material independence of ν_eff_ remains to be quantified. In the second stage, the material and manufacturing route are selected to tune the peak load, the displacement at peak load and the work at a prescribed displacement—the part of the proposition supported by the replicated data. The proposition rests on one architecture, one loading mode and unreplicated optical observations; its generalization requires confirmation with replicated, documented strain measurements on other topologies and loading modes. Within the tested set, Flex 3D represents a compliant regime of low force and low W_14_, whereas PETG, Impact PLA and PLA occupy regimes of higher load and work. Material selection for auxetic lattices should therefore combine the kinematic requirements with application-specific force, displacement and work criteria.

The same two-stage logic can provide a methodological basis for subsequent research within the KOMITMENT project [43]. Architecture-level kinematic descriptors can first be separated from material/process-dependent mechanical descriptors and then combined with simulation and AI-assisted screening. Extending the workflow to basalt-based and other composite systems would allow experimentally obtained descriptors—peak force, displacement at peak load, stiffness extracted with a documented procedure, mechanical work and relevant process variables—to enter datasets for predicting applicability, together with criteria such as mass, durability, environmental stability and life-cycle performance. This link is methodological; the present polymer dataset does not validate KOMITMENT composite formulations.

Application would require further evidence, including specimen mass and relative density, specific energy absorption where relevant, dynamic, cyclic and fatigue testing, environmental conditioning, temperature and moisture sensitivity, and process-control data. The study is therefore a replicated screening benchmark of material/process variants under quasi-static tension, not a validation of impact, crashworthiness or aerospace qualification performance.

## 5. Limitations

Table 7 summarizes the data basis of the study: the measured and archived results on which the conclusions rest and, for each aspect, the resulting scope of interpretation.

Beyond Table 7, the scope of the study is defined as follows. The n = 10 datasets quantify specimen-to-specimen variability within each series; reproducibility between batches, printers, operators or laboratories is a topic for future multi-batch studies. The material-level interpretations in Section 4.2 are qualitative and based on the literature, because polymer morphology, crystallinity, porosity, interlayer bond strength and melt rheology were outside the test program. The sensitivity results of Supplementary Note S1 rest on one digitized curve per variant and show the direction and magnitude of sensitivity rather than statistically supported effects. Dynamic, cyclic, fatigue, environmental, damage-tolerance and long-term process-stability behavior are subjects for further work. A repeat campaign with full raw-data retention, a documented stiffness-fitting procedure and dimensional metrology would allow the PETG–PLA stiffness ranking to be confirmed; until then, the k rankings are reported as findings of the archived dataset.

## 6. Conclusions

- Ninety quasi-static tensile tests (10 specimens for each of nine material/process variants) provided a specimen-level mechanical benchmark for one nominal re-entrant auxetic architecture; the conclusions apply to the tested variants.
- Previously processed optical measurements gave a negative global ν_eff_ (−1.043 to −1.052) for all eight evaluated variants. These descriptive values indicate a consistently auxetic response, whereas the material dependence of ν_eff_ remains to be quantified with replicated strain measurements; the linear FEM model illustrates the auxetic mechanism.
- The material/process variant had a very large effect on all four mechanical metrics (Kruskal–Wallis ε^2^ = 0.924 for F_max_, 0.929 for u(F_max_), 0.942 for k and 0.908 for W_14_), with CV values below 5% within each variant, which indicates specimen-level repeatability rather than batch-level reproducibility.
- PLA had the highest mean F_max_ (68.48 ± 2.40 N). PETG had the highest mean W_14_ (622.80 ± 28.03 N mm), 7.9% above PLA (95% simultaneous CI 1.2–14.5%) despite a 4.3% lower mean F_max_; this ranking applies to the 14 mm displacement limit. PETG also had the highest mean archived stiffness k (8.50 ± 0.36 N/mm), which is interpreted as an archived descriptor (Section 2.7).
- No variant ranked first in all metrics. No statistically significant difference was detected between PETG and Impact PLA in F_max_ or W_14_, while PLA had the highest mean peak load; material selection is therefore a multi-criteria, application-dependent decision.
- PA12 produced by SLS reached an 11.0% higher mean F_max_ and a 21.9% higher mean W_14_ than PA12 produced by FDM, whereas the 3.7% difference in k was not statistically significant. This result characterizes the tested PA12 variants rather than SLS and FDM in general.
- Overall, the data are consistent with a two-stage design proposition in which the architecture provides the auxetic deformation mode and the material/process variant strongly influences the peak load, the displacement at peak load and the work at a prescribed displacement. The second part is supported by the replicated measurements. Establishing the first part quantitatively, and the proposition as a general design strategy, requires replicated and documented strain measurements, further architectures and loading modes, complete process and batch records, dimensional verification, mass and relative-density data, and dynamic, cyclic, fatigue and environmental tests.

## Figures and Tables

**Table 1:** Common FDM slicing configuration of the specimen design and source of each entry.

Parameter	Value	Source
Printer; nozzle	Bambu Lab A1; 0.4 mm	P, C [37]
CAD and slicing software	SolidWorks 2024; Bambu Studio	P, C [37]
Common configuration	Identical printing parameters for the specimens [37]	C [37]
Process profile	“0.20 mm Standard @BBL A1” system profile	P
Layer height (first layer)	0.20 mm (0.20 mm)	P
Line width	Outer wall 0.42 mm; inner wall 0.45 mm; sparse infill 0.45 mm; first layer 0.50 mm	P
Walls	Two loops; classic wall generator; thin-wall detection off; inner/outer wall order	P
Top/bottom solid layers	5/3	P
Sparse infill	15%, grid; solid infill where the sparse area is below 15 mm^2^	P
Nominal speeds	Outer wall 200, inner wall 300, sparse infill 270, first layer 50 mm/s (upper limits, reduced by the volumetric-flow and layer-time limits of the filament profile)	P
Supports; build plate	None; textured polyetherimide (PEI) plate	P
Geometry	176.88 × 80.00 × 5.00 mm; ligaments 1.00 mm; boundary members 2.0 mm; end arms 4.0 mm; 16 node markers 0.75 mm high	P (project mesh)
Orientation	Flat on the build plate; loading direction parallel to the specimen length	P, C [37]
Filament profiles stored	Bambu Lab PLA Basic, ABS and PETG Basic (defaults in Table 2)	D

Note: P, saved in both Bambu Studio projects (re-saved on 9 October 2026); D, filament-profile system default stored in the projects, listed for reference (not a verified printing setting); C, confirmed in the documentation of the original campaign [37]. Material-specific settings are summarized in Table 2.

**Table 2:** Material/process variants, manufacturing route and material designation.

Variant	Route and Configuration	Material Designation	Stored Profile Default, Nozzle/Plate [°C]
PLA	FDM (MEX), Table 1	PLA	220/65 (D)
Impact PLA	FDM (MEX), Table 1	Impact-modified PLA	–
PETG	FDM (MEX), Table 1	PETG	245/70 (D)
ABS	FDM (MEX), Table 1	ABS	270/100 (D)
ASA	FDM (MEX), Table 1	ASA	–
UltraT	FDM (MEX), Table 1	UltraT (trade name)	–
PA12 (FDM)	FDM (MEX), Table 1	PA12	–
PA12 (SLS)	SLS (PBF)	PA12 powder	–
Flex 3D	FDM (MEX), Table 1	Flexible filament (trade name)	–

Note: MEX, material extrusion; PBF, powder bed fusion; D, filament-profile system default stored in the slicer projects, listed for reference only (not the verified printing temperature); –, no entry in the available records. Material designations follow the commercial designations of the filaments and powder (polymer family not verified by chemical analysis). Suppliers, grades, the printing temperatures actually used and the SLS process parameters were not recorded; all conclusions therefore refer to the tested variants.

**Table 3:** Global effective Poisson’s ratio ν_eff_ from optical marker tracking (one previously processed value per variant; descriptive values, Section 2.5). PA12 (SLS) was not evaluated optically; the values for PETG and Flex 3D come from a later evaluation with white node markers (Section 2.5).

Material	Optical ν_eff_ [−]	Source
PLA	−1.050	Reported in [37]
Impact PLA	−1.047	Reported in [37]
PETG	−1.047	Later evaluation, white markers (Section 2.5)
ABS	−1.052	Reported in [37]
ASA	−1.046	Reported in [37]
UltraT	−1.049	Reported in [37]
PA12 (FDM)	−1.044	Reported in [37]
Flex 3D	−1.043	Later evaluation, white markers (Section 2.5)

**Table 4:** Mechanical metrics of the tested specimens (mean ± SD, n = 10 per variant). F_max_, maximum force; u(F_max_), crosshead displacement at peak load; k, archived structural stiffness (Section 2.7); W_14_, mechanical work up to 14 mm crosshead displacement; W_rel_, relative mechanical work (Equation (3)).

Material	F_max_ [N]	u(F_max_) [mm]	k [N/mm]	W_14_ [N mm]	W_14_ [J]	W_rel_ [%]
PLA	68.48 ± 2.40	14.10 ± 0.25	7.08 ± 0.29	577.46 ± 19.29	0.577 ± 0.019	92.72
Impact PLA	65.86 ± 2.07	13.54 ± 0.28	7.58 ± 0.21	590.78 ± 16.96	0.591 ± 0.017	94.86
PETG	65.52 ± 2.11	10.91 ± 0.20	8.50 ± 0.36	622.80 ± 28.03	0.623 ± 0.028	100.00
ABS	50.01 ± 1.79	11.32 ± 0.14	5.63 ± 0.19	451.53 ± 15.85	0.452 ± 0.016	72.50
ASA	51.52 ± 1.94	8.88 ± 0.23	6.90 ± 0.22	449.61 ± 20.02	0.450 ± 0.020	72.19
UltraT	62.21 ± 1.41	14.00 ± 0.26	6.87 ± 0.28	572.12 ± 19.77	0.572 ± 0.020	91.86
PA12 (FDM)	52.31 ± 1.40	16.41 ± 0.47	4.97 ± 0.15	355.50 ± 11.99	0.355 ± 0.012	57.08
PA12 (SLS)	58.05 ± 0.91	14.04 ± 0.22	5.16 ± 0.11	433.26 ± 16.76	0.433 ± 0.017	69.57
Flex 3D	25.89 ± 0.93	15.64 ± 0.46	1.63 ± 0.06	187.18 ± 8.79	0.187 ± 0.009	30.06

**Table 5:** Omnibus tests and Kruskal–Wallis effect sizes ε^2^ for the four mechanical metrics.

Metric	Primary Omnibus Test	Statistic (df)	*p*	Robust ε^2^
F_max_ [N]	One-way ANOVA	F = 564.25 (8, 81)	<0.001	0.924
u(F_max_) [mm]	Welch ANOVA	F = 687.32 (8, 33.48)	<0.001	0.929
k [N/mm]	Welch ANOVA	F = 2686.34 (8, 32.80)	<0.001	0.942
W_14_ [N mm]	Welch ANOVA	F = 1049.21 (8, 33.45)	<0.001	0.908

**Table 6:** Principal pairwise contrasts with 95% simultaneous confidence intervals and Hedges’ g (n = 10 per group).

Metric	Contrast (A − B)	Mean Difference (95% CI)	Relative to B [%]	Adjusted *p*	Hedges’ g
F_max_ [N]	PETG − PLA	−2.96 (−5.44, −0.49)	−4.3	0.008	−1.26
F_max_ [N]	Impact PLA − PLA	−2.62 (−5.10, −0.15)	−3.8	0.029	−1.12
F_max_ [N]	PETG − Impact PLA	−0.34 (−2.81, 2.13)	−0.5	1.000	−0.16
F_max_ [N]	PA12 (SLS) − PA12 (FDM)	5.74 (3.26, 8.21)	11.0	<0.001	4.67
u(F_max_) [mm]	PETG − PLA	−3.19 (−3.55, −2.83)	−22.6	<0.001	−13.30
u(F_max_) [mm]	PA12 (SLS) − PA12 (FDM)	−2.37 (−2.97, −1.77)	−14.4	<0.001	−6.22
k [N/mm]	PETG − PLA	1.42 (0.90, 1.93)	20.0	<0.001	4.15
k [N/mm]	PA12 (SLS) − PA12 (FDM)	0.19 (−0.02, 0.39)	3.7	0.090	1.38
W_14_ [N mm]	PETG − PLA	45.34 (7.04, 83.63)	7.9	0.014	1.80
W_14_ [N mm]	PETG − Impact PLA	32.02 (−5.25, 69.29)	5.4	0.121	1.32
W_14_ [N mm]	Impact PLA − PLA	13.31 (−15.21, 41.84)	2.3	0.773	0.70
W_14_ [N mm]	PA12 (SLS) − PA12 (FDM)	77.77 (54.64, 100.89)	21.9	<0.001	5.11

Note: Differences are A − B in the units of the metric; relative differences refer to the mean of B. Confidence intervals are simultaneous 95% intervals of Tukey’s HSD test (F_max_) or the Games–Howell procedure (u(F_max_), k, W_14_) across all 36 pairs and agree with the adjusted *p*-values in Supplementary Tables S2–S5. Hedges’ g is the mean difference divided by the pooled SD of the two groups, corrected for small-sample bias; its very large values reflect the low within-group scatter and do not indicate process-level reproducibility. k is the archived structural stiffness, whose contrasts depended strongly on the fitting window on the single-specimen curves (Section 2.7, Supplementary Note S1). W_14_ depends on the 14 mm limit.

**Table 7:** Data basis of the study and scope of interpretation.

Aspect	Data Basis	Use and Scope of Interpretation
F_max_ and u(F_max_)	Measured for all 90 specimens (Table S1)	Basis of all inferential analyses
Structural stiffness k	Archived values for all 90 specimens; fitting window documented for a reference PLA test (2–8 mm) [37]	Archived descriptor; window sensitivity up to 28% (Note S1)
Mechanical work W_14_	Archived values for all 90 specimens; common 14 mm limit [37]	Rankings refer to the 14 mm limit (Note S1)
Force–displacement time series	Stored at the former workplace and not accessible for re-analysis; single-specimen curves digitized from [37]	Results based on scalar metrics (Table S1); digitized curves used for sensitivity analysis only (Note S1)
Effective Poisson’s ratio ν_eff_	One previously processed optical value per variant (Table 3); evaluation script not available; idealized FEM model (Table S8)	Descriptive; no accuracy value assigned; optical and FEM results presented separately
Force measurement	CHCONTECH CH-SD-500kg load cell (4.90 kN rated); peak forces 0.5–1.5% of capacity; no calibration certificate for the test period	Comparative use within the test series
Manufacturing	Slicer projects of the design (Table 1); stored profile defaults and commercial designations (Table 2); print-job settings not recorded	Conclusions refer to the tested material/process variants
PA12 FDM–SLS	Two replicated groups (n = 10 each)	Material–process contrast
Replication	Ten specimens per variant	Specimen-level replication; batch effects beyond scope
Geometry and mass	Nominal CAD geometry (Section 2.1); specimen dimensions and mass not measured	Combined material–process–geometry effects; mass-specific metrics beyond scope
Failure and environment	Indoor laboratory conditions; temperature, humidity and failure modes not recorded	Failure modes and moisture effects beyond scope

## Data Availability

The specimen-level dataset used for all statistical analyses is provided in Supplementary Table S1, and the adjusted pairwise comparisons are provided in Supplementary Tables S2–S5. The machine-export force–displacement time series of the 90 tests are stored on the testing-machine computer at the authors’ former workplace and were not accessible for this revision; no reconstructed curves are provided. The digitized single-specimen curves used in Supplementary Note S1 are derived from a plotted figure in [37]. The Python script used for the original optical evaluation is no longer available to the authors, and the optical values were not re-analyzed. The Bambu Studio project files of the specimen design are available from the corresponding author on reasonable request. The availability of all other records is summarized in Table 7.

## Supplementary Materials

The following supporting information can be downloaded at: https://www.mdpi.com/article/10.3390/ma19214506/s1, Table S1, complete specimen-level dataset of the 90 tensile tests; Tables S2–S5, adjusted pairwise comparisons for Fmax, u(Fmax), k and W14; Supplementary Note S1, admissibility analysis of the retained metrics and sensitivity analysis on digitized single-specimen curves, with Figures S1 and S2 and Tables S6 and S7; Table S8, global νeff of the idealized linear FEM model.

## Abbreviations and Symbols

The following abbreviations and symbols are used in this manuscript.

Abbreviation or symbol	Meaning
ABS	Acrylonitrile butadiene styrene
AI	Artificial intelligence
AM	Additive manufacturing
ANOVA	Analysis of variance
ASA	Acrylonitrile styrene acrylate
CAD	Computer-aided design
CI	Confidence interval
CMOS	Complementary metal–oxide–semiconductor
CV	Coefficient of variation
DIC	Digital image correlation
FDM	Fused deposition modeling
FEM	Finite-element method
HDF5	Hierarchical Data Format version 5
HSD	Honestly significant difference (Tukey)
MEX	Material extrusion
PA12	Polyamide 12
PBF	Powder bed fusion
PEI	Polyetherimide
PETG	Glycol-modified polyethylene terephthalate
PLA	Polylactic acid
SD	Standard deviation
SLS	Selective laser sintering
YOLO	You Only Look Once (object-detection model)
F_max_	Maximum force [N]
u	Crosshead displacement [mm]
u(F_max_)	Crosshead displacement at peak load [mm]
k	Archived structural stiffness [N/mm]
F(u)	Force at crosshead displacement u [N]
W_14_	Mechanical work up to u = 14 mm [N mm]
W_i_, W_max_	Mean W_14_ of variant i; highest group mean of W_14_ [N mm]
W_rel_	Relative mechanical work [%]
ν_eff_	Effective Poisson’s ratio of the lattice [−]
ε_ax_, ε_trans_	Axial and transverse engineering strains of the tracked region [−]
L_0_, W_0_	Initial axial and transverse dimensions of the evaluated region [mm]
ΔL, ΔW	Changes in L_0_ and W_0_ during loading [mm]
E, ν	Young’s modulus [MPa] and Poisson’s ratio of an isotropic material model
E_x_, E_y_, E_z_; G_xy_, G_yz_, G_zx_; ν_xy_, ν_yz_, ν_zx_	Young’s moduli, shear moduli [MPa] and Poisson’s ratios of the orthotropic model
θ, t, h, w	Unit-cell angle [°], ligament width, vertex distance and cell width [mm]
n, N	Specimens per variant; total number of specimens
H, m, ε^2^	Kruskal–Wallis statistic, number of groups and effect size
g	Hedges’ standardized effect size
p, α	*p*-value and significance level
