# Supplementary material for: Beyond Poisson’s Ratio: Material–Process Performance of Additively Manufactured Auxetic Lattices
Source: Materials (Basel). 2026 Oct 26;19(21):4506. doi: 10.3390/ma19214506 (PMC13763948; doi:10.3390/ma19214506)
Supplement: Supplementary file 1 [file materials-19-04506-s001.zip › materials-4605029-supplementary.pdf]

materials
MDPI

Supplementary Materials

Beyond Poisson's Ratio: Material–Process Performance of Additively Manufactured Auxetic Lattices

Barbara Schürger[1]; Michal Hovanec[1]; Peter Palička[1] and Robert Szaniszló[2]

Contents. This file contains the supporting information of the article: Table S1 (specimen-level mechanical dataset), Tables S2–S5 (adjusted pairwise comparisons), Supplementary Note S1 with Figures S1 and S2 and Tables S6 and S7, and Table S8 (idealized finite-element results). References numbered without the prefix "S", including reference [37], correspond to the bibliography of the main article.

Table S1. Specimen-level mechanical dataset (10 specimens per material/process variant; N = 90).

Material	Specimen	F_{max} [N]	$u(F_{max})$ [mm]	k [N/mm]	W_{14} [Nmm]
PLA	1	67.84	13.86	7.33	600.54
PLA	2	66.47	13.94	7.04	574.60
PLA	3	66.40	14.07	6.52	555.02
PLA	4	69.24	14.17	7.32	579.61
PLA	5	65.39	14.46	6.71	553.55
PLA	6	69.85	14.03	6.93	585.82
PLA	7	70.19	14.61	7.17	601.72
PLA	8	65.89	13.81	7.38	552.35
PLA	9	71.95	14.01	7.31	598.05
PLA	10	71.56	14.07	7.12	573.37
Impact PLA	1	66.83	13.66	7.65	579.60
Impact PLA	2	69.91	13.21	7.76	619.76
Impact PLA	3	62.65	13.49	7.25	590.01
Impact PLA	4	65.54	13.59	7.58	580.86
Impact PLA	5	65.26	13.69	7.79	592.90
Impact PLA	6	67.75	13.08	7.69	608.78
Impact PLA	7	64.68	13.92	7.33	576.05
Impact PLA	8	63.82	13.35	7.27	561.97
Impact PLA	9	66.90	13.48	7.68	601.90
Impact PLA	10	65.21	13.93	7.75	595.94
PETG	1	67.08	10.95	8.38	615.56
PETG	2	68.33	10.95	8.57	652.55
PETG	3	61.89	10.99	7.91	584.77
PETG	4	66.41	10.61	8.85	638.86
PETG	5	63.32	11.38	7.89	589.12
PETG	6	65.39	10.87	8.45	611.59
PETG	7	66.05	10.69	8.54	594.09
PETG	8	63.05	10.94	8.68	634.75
PETG	9	66.05	10.88	8.78	640.35
PETG	10	67.58	10.87	8.94	666.34
ABS	1	50.52	11.44	5.68	463.79
ABS	2	52.59	11.26	5.79	467.56
ABS	3	50.53	11.20	5.81	449.30

Material	Specimen	F_{max} [N]	$u(F_{max})$ [mm]	k [N/mm]	W_{14} [Nmm]
ABS	4	49.57	11.19	5.70	436.84
ABS	5	46.32	11.35	5.41	442.44
ABS	6	50.62	11.18	5.71	453.48
ABS	7	48.25	11.41	5.27	418.72
ABS	8	49.01	11.39	5.49	449.70
ABS	9	51.22	11.22	5.84	468.26
ABS	10	51.43	11.60	5.57	465.23
ASA	1	55.62	8.57	7.09	477.85
ASA	2	51.85	8.62	7.33	476.97
ASA	3	50.92	8.99	6.82	457.70
ASA	4	49.03	9.03	6.76	454.73
ASA	5	49.89	8.65	6.96	429.70
ASA	6	53.88	9.14	7.10	464.48
ASA	7	51.14	9.10	6.87	433.00
ASA	8	50.63	8.85	6.54	421.83
ASA	9	51.84	9.15	6.78	447.55
ASA	10	50.40	8.72	6.76	432.25
UltraT	1	63.32	14.09	7.07	593.88
UltraT	2	62.13	13.90	6.66	568.59
UltraT	3	60.87	14.02	6.85	580.41
UltraT	4	60.33	14.27	6.56	549.23
UltraT	5	64.01	13.89	7.14	586.29
UltraT	6	63.30	13.79	6.91	558.90
UltraT	7	62.03	13.42	7.24	604.11
UltraT	8	61.21	14.27	6.68	544.40
UltraT	9	60.76	14.21	6.44	556.94
UltraT	10	64.12	14.13	7.16	578.44
PA12 (FDM)	1	51.79	16.84	4.86	334.64
PA12 (FDM)	2	54.62	16.04	5.08	370.73
PA12 (FDM)	3	50.20	16.93	4.94	346.56
PA12 (FDM)	4	52.97	16.41	5.09	363.74
PA12 (FDM)	5	54.05	15.87	5.12	361.54
PA12 (FDM)	6	51.84	16.13	4.73	352.61
PA12 (FDM)	7	52.51	16.12	5.14	350.20
PA12 (FDM)	8	50.65	17.32	4.79	344.58
PA12 (FDM)	9	51.55	16.11	4.91	372.21
PA12 (FDM)	10	52.96	16.36	5.09	358.16
PA12 (SLS)	1	58.17	13.80	5.06	404.56
PA12 (SLS)	2	56.69	13.91	5.16	426.04
PA12 (SLS)	3	58.92	13.79	5.33	446.06
PA12 (SLS)	4	58.23	13.79	5.20	411.09
PA12 (SLS)	5	59.22	14.30	5.15	443.19
PA12 (SLS)	6	58.44	13.99	5.23	457.11
PA12 (SLS)	7	56.68	14.22	5.06	423.92
PA12 (SLS)	8	57.29	14.06	5.06	435.97
PA12 (SLS)	9	57.94	14.37	5.05	436.40
PA12 (SLS)	10	58.93	14.19	5.31	448.30
Flex 3D	1	26.80	15.66	1.64	187.74
Flex 3D	2	25.18	15.77	1.63	190.68
Flex 3D	3	24.98	15.18	1.56	197.89
Flex 3D	4	25.09	16.15	1.60	184.50
Flex 3D	5	25.52	15.62	1.64	190.51

https://doi.org/10.3390/ma19214506

Material	Specimen	F_{max} [N]	$u(F_{max})$ [mm]	k [N/mm]	W_{14} [Nmm]
Flex 3D	6	27.60	15.03	1.72	193.78
Flex 3D	7	24.78	16.46	1.59	177.97
Flex 3D	8	26.45	15.92	1.70	186.31
Flex 3D	9	26.46	15.47	1.52	168.00
Flex 3D	10	25.99	15.12	1.68	194.45

Supplementary Statistical Pairwise Comparisons

Table S2. Tukey's HSD pairwise comparisons for the maximum force F_{max}.

Material/process pair	Mean difference [N]	Adjusted p	Significant	Method
PLA – Impact PLA	2.623	0.029	Yes	Tukey HSD
PLA – PETG	2.963	0.008	Yes	Tukey HSD
PLA – ABS	18.472	<0.001	Yes	Tukey HSD
PLA – ASA	16.958	<0.001	Yes	Tukey HSD
PLA – UltraT	6.270	<0.001	Yes	Tukey HSD
PLA – PA12 (FDM)	16.164	<0.001	Yes	Tukey HSD
PLA – PA12 (SLS)	10.427	<0.001	Yes	Tukey HSD
PLA – Flex 3D	42.593	<0.001	Yes	Tukey HSD
Impact PLA – PETG	0.340	1.000	No	Tukey HSD
Impact PLA – ABS	15.849	<0.001	Yes	Tukey HSD
Impact PLA – ASA	14.335	<0.001	Yes	Tukey HSD
Impact PLA – UltraT	3.647	<0.001	Yes	Tukey HSD
Impact PLA – PA12 (FDM)	13.541	<0.001	Yes	Tukey HSD
Impact PLA – PA12 (SLS)	7.804	<0.001	Yes	Tukey HSD
Impact PLA – Flex 3D	39.970	<0.001	Yes	Tukey HSD
PETG – ABS	15.509	<0.001	Yes	Tukey HSD
PETG – ASA	13.995	<0.001	Yes	Tukey HSD
PETG – UltraT	3.307	0.002	Yes	Tukey HSD
PETG – PA12 (FDM)	13.201	<0.001	Yes	Tukey HSD
PETG – PA12 (SLS)	7.464	<0.001	Yes	Tukey HSD
PETG – Flex 3D	39.630	<0.001	Yes	Tukey HSD
ABS – ASA	-1.514	0.582	No	Tukey HSD
ABS – UltraT	-12.202	<0.001	Yes	Tukey HSD
ABS – PA12 (FDM)	-2.308	0.087	No	Tukey HSD
ABS – PA12 (SLS)	-8.045	<0.001	Yes	Tukey HSD
ABS – Flex 3D	24.121	<0.001	Yes	Tukey HSD
ASA – UltraT	-10.688	<0.001	Yes	Tukey HSD
ASA – PA12 (FDM)	-0.794	0.983	No	Tukey HSD
ASA – PA12 (SLS)	-6.531	<0.001	Yes	Tukey HSD
ASA – Flex 3D	25.635	<0.001	Yes	Tukey HSD
UltraT – PA12 (FDM)	9.894	<0.001	Yes	Tukey HSD
UltraT – PA12 (SLS)	4.157	<0.001	Yes	Tukey HSD
UltraT – Flex 3D	36.323	<0.001	Yes	Tukey HSD
PA12 (FDM) – PA12 (SLS)	-5.737	<0.001	Yes	Tukey HSD
PA12 (FDM) – Flex 3D	26.429	<0.001	Yes	Tukey HSD
PA12 (SLS) – Flex 3D	32.166	<0.001	Yes	Tukey HSD

Note: Mean difference is calculated as the first-listed group mean minus the second-listed group mean. Adjusted p-values use the post hoc method specified in the last column; significance

threshold α = 0.05. These comparisons are based on the 10 physical specimens per material/process variant in Supplementary Table S1.

Table S3. Games–Howell pairwise comparisons for the crosshead displacement at peak load u(F_{max}).

Material/process pair	Mean difference [mm]	Adjusted p	Significant	Method
PLA − Impact PLA	0.563	0.004	Yes	Games–Howell
PLA − PETG	3.190	<0.001	Yes	Games–Howell
PLA − ABS	2.779	<0.001	Yes	Games–Howell
PLA − ASA	5.221	<0.001	Yes	Games–Howell
PLA − UltraT	0.104	0.990	No	Games–Howell
PLA − PA12 (FDM)	-2.310	<0.001	Yes	Games–Howell
PLA − PA12 (SLS)	0.061	1.000	No	Games–Howell
PLA − Flex 3D	-1.535	<0.001	Yes	Games–Howell
Impact PLA − PETG	2.627	<0.001	Yes	Games–Howell
Impact PLA − ABS	2.216	<0.001	Yes	Games–Howell
Impact PLA − ASA	4.658	<0.001	Yes	Games–Howell
Impact PLA − UltraT	-0.459	0.028	Yes	Games–Howell
Impact PLA − PA12 (FDM)	-2.873	<0.001	Yes	Games–Howell
Impact PLA − PA12 (SLS)	-0.502	0.008	Yes	Games–Howell
Impact PLA − Flex 3D	-2.098	<0.001	Yes	Games–Howell
PETG − ABS	-0.411	0.002	Yes	Games–Howell
PETG − ASA	2.031	<0.001	Yes	Games–Howell
PETG − UltraT	-3.086	<0.001	Yes	Games–Howell
PETG − PA12 (FDM)	-5.500	<0.001	Yes	Games–Howell
PETG − PA12 (SLS)	-3.129	<0.001	Yes	Games–Howell
PETG − Flex 3D	-4.725	<0.001	Yes	Games–Howell
ABS − ASA	2.442	<0.001	Yes	Games–Howell
ABS − UltraT	-2.675	<0.001	Yes	Games–Howell
ABS − PA12 (FDM)	-5.089	<0.001	Yes	Games–Howell
ABS − PA12 (SLS)	-2.718	<0.001	Yes	Games–Howell
ABS − Flex 3D	-4.314	<0.001	Yes	Games–Howell
ASA − UltraT	-5.117	<0.001	Yes	Games–Howell
ASA − PA12 (FDM)	-7.531	<0.001	Yes	Games–Howell
ASA − PA12 (SLS)	-5.160	<0.001	Yes	Games–Howell
ASA − Flex 3D	-6.756	<0.001	Yes	Games–Howell
UltraT − PA12 (FDM)	-2.414	<0.001	Yes	Games–Howell
UltraT − PA12 (SLS)	-0.043	1.000	No	Games–Howell
UltraT − Flex 3D	-1.639	<0.001	Yes	Games–Howell
PA12 (FDM) − PA12 (SLS)	2.371	<0.001	Yes	Games–Howell
PA12 (FDM) − Flex 3D	0.775	0.032	Yes	Games–Howell
PA12 (SLS) − Flex 3D	-1.596	<0.001	Yes	Games–Howell

Note: Mean difference is calculated as the first-listed group mean minus the second-listed group mean. Adjusted p-values use the post hoc method specified in the last column; significance threshold α = 0.05. These comparisons are based on the 10 physical specimens per material/process variant in Supplementary Table S1.

Table S4. Games–Howell pairwise comparisons for the archived structural stiffness k.

Material/process pair	Mean difference [N/mm]	Adjusted p	Significant	Method
PLA − Impact PLA	-0.492	0.010	Yes	Games–Howell
PLA − PETG	-1.416	<0.001	Yes	Games–Howell

PLA − ABS	1.456	<0.001	Yes	Games–Howell
PLA − ASA	0.182	0.805	No	Games–Howell
PLA − UltraT	0.212	0.754	No	Games–Howell
PLA − PA12 (FDM)	2.108	<0.001	Yes	Games–Howell
PLA − PA12 (SLS)	1.922	<0.001	Yes	Games–Howell
PLA − Flex 3D	5.455	<0.001	Yes	Games–Howell
Impact PLA − PETG	-0.924	<0.001	Yes	Games–Howell
Impact PLA − ABS	1.948	<0.001	Yes	Games–Howell
Impact PLA − ASA	0.674	<0.001	Yes	Games–Howell
Impact PLA − UltraT	0.704	<0.001	Yes	Games–Howell
Impact PLA − PA12 (FDM)	2.600	<0.001	Yes	Games–Howell
Impact PLA − PA12 (SLS)	2.414	<0.001	Yes	Games–Howell
Impact PLA − Flex 3D	5.947	<0.001	Yes	Games–Howell
PETG − ABS	2.872	<0.001	Yes	Games–Howell
PETG − ASA	1.598	<0.001	Yes	Games–Howell
PETG − UltraT	1.628	<0.001	Yes	Games–Howell
PETG − PA12 (FDM)	3.524	<0.001	Yes	Games–Howell
PETG − PA12 (SLS)	3.338	<0.001	Yes	Games–Howell
PETG − Flex 3D	6.871	<0.001	Yes	Games–Howell
ABS − ASA	-1.274	<0.001	Yes	Games–Howell
ABS − UltraT	-1.244	<0.001	Yes	Games–Howell
ABS − PA12 (FDM)	0.652	<0.001	Yes	Games–Howell
ABS − PA12 (SLS)	0.466	<0.001	Yes	Games–Howell
ABS − Flex 3D	3.999	<0.001	Yes	Games–Howell
ASA − UltraT	0.030	1.000	No	Games–Howell
ASA − PA12 (FDM)	1.926	<0.001	Yes	Games–Howell
ASA − PA12 (SLS)	1.740	<0.001	Yes	Games–Howell
ASA − Flex 3D	5.273	<0.001	Yes	Games–Howell
UltraT − PA12 (FDM)	1.896	<0.001	Yes	Games–Howell
UltraT − PA12 (SLS)	1.710	<0.001	Yes	Games–Howell
UltraT − Flex 3D	5.243	<0.001	Yes	Games–Howell
PA12 (FDM) − PA12 (SLS)	-0.186	0.090	No	Games–Howell
PA12 (FDM) − Flex 3D	3.347	<0.001	Yes	Games–Howell
PA12 (SLS) − Flex 3D	3.533	<0.001	Yes	Games–Howell

Note: Mean difference is calculated as the first-listed group mean minus the second-listed group mean. Adjusted p-values use the post hoc method specified in the last column; significance threshold $\alpha = 0.05$. These comparisons are based on the 10 physical specimens per material/process variant in Supplementary Table S1.

Table S5. Games–Howell pairwise comparisons for the mechanical work W_{14}.

**Material/process pair**	**Mean difference [N mm]**	**Adjusted p**	**Significant**	**Method**
PLA − Impact PLA	-13.314	0.773	No	Games–Howell
PLA − PETG	-45.335	0.014	Yes	Games–Howell
PLA − ABS	125.931	<0.001	Yes	Games–Howell
PLA − ASA	127.857	<0.001	Yes	Games–Howell
PLA − UltraT	5.344	0.999	No	Games–Howell
PLA − PA12 (FDM)	221.966	<0.001	Yes	Games–Howell
PLA − PA12 (SLS)	144.199	<0.001	Yes	Games–Howell
PLA − Flex 3D	390.280	<0.001	Yes	Games–Howell
Impact PLA − PETG	-32.021	0.121	No	Games–Howell

https://doi.org/10.3390/ma19214506

Impact PLA − ABS	139.245	<0.001	Yes	Games–Howell
Impact PLA − ASA	141.171	<0.001	Yes	Games–Howell
Impact PLA − UltraT	18.658	0.410	No	Games–Howell
Impact PLA − PA12 (FDM)	235.280	<0.001	Yes	Games–Howell
Impact PLA − PA12 (SLS)	157.513	<0.001	Yes	Games–Howell
Impact PLA − Flex 3D	403.594	<0.001	Yes	Games–Howell
PETG − ABS	171.266	<0.001	Yes	Games–Howell
PETG − ASA	173.192	<0.001	Yes	Games–Howell
PETG − UltraT	50.679	0.006	Yes	Games–Howell
PETG − PA12 (FDM)	267.301	<0.001	Yes	Games–Howell
PETG − PA12 (SLS)	189.534	<0.001	Yes	Games–Howell
PETG − Flex 3D	435.615	<0.001	Yes	Games–Howell
ABS − ASA	1.926	1.000	No	Games–Howell
ABS − UltraT	-120.587	<0.001	Yes	Games–Howell
ABS − PA12 (FDM)	96.035	<0.001	Yes	Games–Howell
ABS − PA12 (SLS)	18.268	0.292	No	Games–Howell
ABS − Flex 3D	264.349	<0.001	Yes	Games–Howell
ASA − UltraT	-122.513	<0.001	Yes	Games–Howell
ASA − PA12 (FDM)	94.109	<0.001	Yes	Games–Howell
ASA − PA12 (SLS)	16.342	0.575	No	Games–Howell
ASA − Flex 3D	262.423	<0.001	Yes	Games–Howell
UltraT − PA12 (FDM)	216.622	<0.001	Yes	Games–Howell
UltraT − PA12 (SLS)	138.855	<0.001	Yes	Games–Howell
UltraT − Flex 3D	384.936	<0.001	Yes	Games–Howell
PA12 (FDM) − PA12 (SLS)	-77.767	<0.001	Yes	Games–Howell
PA12 (FDM) − Flex 3D	168.314	<0.001	Yes	Games–Howell
PA12 (SLS) − Flex 3D	246.081	<0.001	Yes	Games–Howell

Note: Mean difference is calculated as the first-listed group mean minus the second-listed group mean. Adjusted p-values use the post hoc method specified in the last column; significance threshold $\alpha = 0.05$. These comparisons are based on the 10 physical specimens per material/process variant in Supplementary Table S1.

Supplementary Note S1. Admissibility and sensitivity analysis of the retained force–displacement metrics

Purpose and data. The 90 tests are represented only by four scalars per specimen (Table S1). This note examines (i) whether these scalars determine the underlying force–displacement curve and (ii) how sensitive k and W are to the choices of fitting window and displacement limit. Item (ii) uses the only force–displacement curves of this specimen design that are currently accessible: one experimental curve per material/process variant, recorded with the same geometry and test procedure and reported as a plotted figure in [37]. Their relationship to the 90 specimens of Table S1 has not been established: their scalar values lie within the ranges of the corresponding groups in Table S1 but coincide with none of the individual specimens. The digitized curves are therefore treated as illustrative experimental records, not as raw machine exports of the tested series; the curves in this note are either such digitized records (Figure S1) or mathematical constructions, and no inferential statistic uses them.

Digitization. The curves were extracted from the raster figure by color segmentation and continuity tracking, with axes calibrated on the plotted grid lines (resolution approximately 0.017 mm and 0.11 N per pixel), and resampled at 0.01 mm. The procedure assumes a linear axis scaling and resolves each pixel column to one force value; segments hidden by overlapping curves or by gaps in dashed lines were bridged by linear

interpolation, and vertical load drops are represented within one pixel column. Compared with the archived scalar values reported for these curves in [37], the digitized curves reproduced F_{max} within 0.42%, $u(F_{max})$ within 0.32%, and W_{14} within 0.58% (trapezoidal integration) for all nine variants (Table S6). The archived k was reproduced by a 2–8 mm window only for PLA and ABS (within 0.2%); for the other variants the 2–8 mm slope differed by −20.7% to +19.9%, and for PA12 (FDM) and PA12 (SLS) the archived value exceeded the steepest regression slope over any 3 mm window.

Admissibility of the scalars. Let F(u) be sampled on a 0.05 mm grid. A curve is admissible if F(0) = 0, F is non-decreasing up to $u(F_{max})$, $F(u(F_{max})) = F_{max}$ and $F \leq F_{max}$, F is non-increasing after the peak, the least-squares slope on 2–8 mm equals k with deviations from linearity within 3% of F_{max}, and F is concave between 8 mm and the peak. These constraints are linear, so the minimum and maximum of W_{14} over all admissible curves follow from linear programming. For the group means, the admissible W_{14} interval spans 47–110% of the measured W_{14} (Table S7), so F_{max}, $u(F_{max})$, and k do not determine W_{14}. When W_{14} is also prescribed, the force at 6 mm can still vary by 8.0–14.8 N between admissible curves, so the four scalars do not determine the curve shape. All 90 specimen tuples in Table S1 are admissible, which is a necessary consistency condition but not a verification. A one-parameter monotone power-law curve $F = F_{max}[1 - (1 - u/u(F_{max}))^n]$ cannot match k and W_{14} simultaneously (e.g., implied W_{14} of 674 vs. measured 577 N mm for PLA), indicating that the real responses contain the initial seating region visible in Figure S1.

Reconstruction test. Applying a minimum-curvature admissible reconstruction to the scalars of each digitized curve and comparing the result with the digitized curve gave maximum pointwise errors of 1–8% of F_{max} for seven variants and 26% for ASA, whose response contains abrupt load drops after the peak; for UltraT the error was 4%, but the true curve itself violates the admissibility assumptions because it contains a load drop before the peak. Reconstructed curves can therefore illustrate only smooth responses and cannot recover damage-related features; they are not used in the main text.

Sensitivity of k and W. On the digitized curves, k changed by 7–28% within a single curve across six windows between 1 and 9 mm (Table S6). PETG had the highest k for every window, but its advantage over PLA ranged from 1% (3–9 mm) to 23% (1–5 mm) (Figure S2a). Between PA12 (SLS) and PA12 (FDM), uniformly extracted k differed by 15–27% for every window, compared with 5% between the archived values of these curves and 3.7% between the archived group means of Table S1. PETG also had the highest mechanical work at every displacement limit between 6 and 16 mm, but its advantage over PLA decreased from 23% at 6 mm to 7% at 14 mm and 3% at 16 mm (Figure S2b); the post-peak part of the interval contributed 31% of W_{14} for PETG and 49% for ASA, and the order of ABS, ASA, and PA12 (SLS) changed above 14 mm. With one curve per variant and an unestablished relationship to the tested series, these results illustrate the direction and magnitude of sensitivity; they are not generalized to the 90 specimens and do not support statistical conclusions.

https://doi.org/10.3390/ma19214506

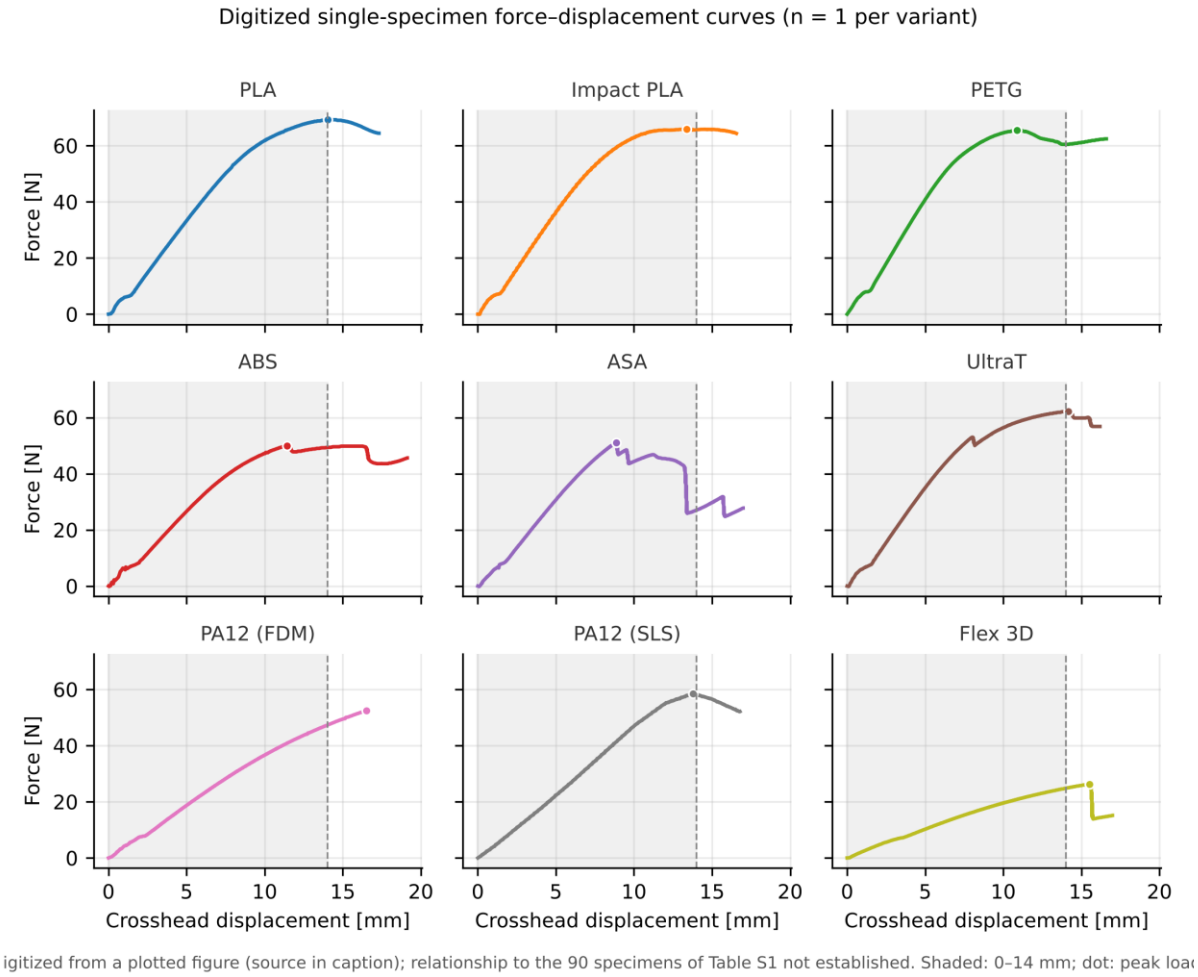

Figure S1. Experimental single-specimen force–displacement curves (one per variant) digitized from a plotted figure in [37]; the curves share the specimen design and test procedure with the 90 tests of Table S1, but their relationship to these specimens is not known; they are used for sensitivity analysis only. Shading marks the 0–14 mm interval used for W_{14}; dots mark the peak load.

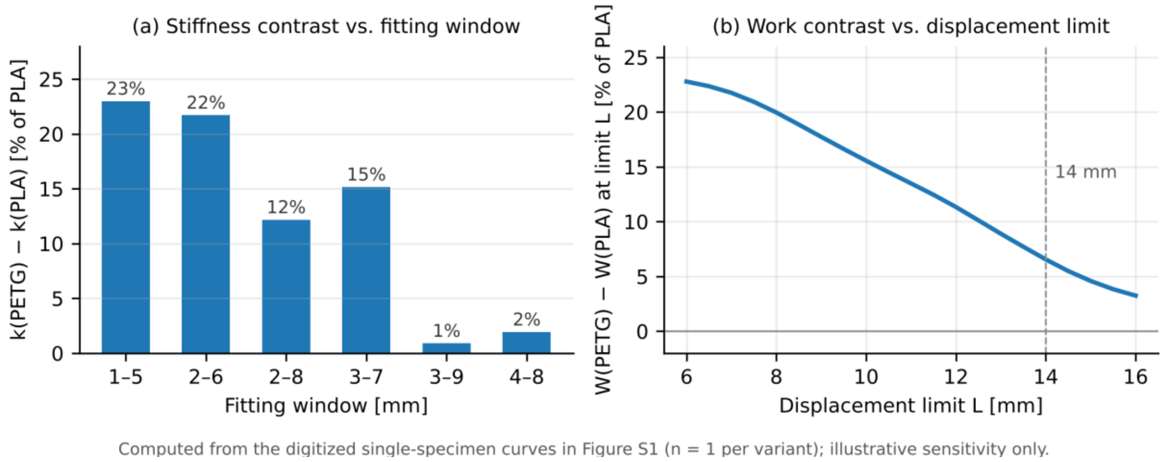

Figure S2. Sensitivity of the PETG–PLA contrasts computed from the digitized curves in Figure S1: (a) difference in k for six linear-fit windows; (b) difference in mechanical work for displacement limits between 6 and 16 mm. One specimen per variant; illustrative sensitivity only.

Table S6. Digitized single-specimen curves compared with the archived scalar values reported for them in [37], and sensitivity of k to the fitting window (windows 1–5, 2–6, 2–8, 3–7, 3–9, and 4–8 mm).

Variant	F_{max} [N] archived / digitized	$u(F_{max})$ [mm] archived / digitized	W_{14} [N mm] archived / digitized	k [N/mm] archived	k(2–8 mm) digitized	k range over six windows
PLA	69.20 / 69.21	14.05 / 14.04	588.1 / 588.8	7.09	7.08	6.71–7.35
Impact PLA	65.84 / 65.88	13.39 / 13.36	601.0 / 604.4	7.67	7.43	6.74–7.98
PETG	65.40 / 65.41	10.92 / 10.89	626.4 / 627.4	8.50	7.94	6.77–8.99
ABS	50.00 / 49.99	11.45 / 11.44	449.0 / 449.8	5.53	5.52	5.06–5.90
ASA	51.06 / 51.11	8.86 / 8.87	454.4 / 454.4	6.89	6.35	5.79–6.76
UltraT	62.20 / 62.24	14.12 / 14.15	561.2 / 561.9	6.83	7.07	5.88–7.70
PA12 (FDM)	52.48 / 52.51	16.52 / 16.53	354.2 / 354.5	4.96	3.93	3.67–4.01
PA12 (SLS)	58.45 / 58.49	13.83 / 13.79	440.6 / 442.3	5.21	4.75	4.55–4.88
Flex 3D	26.40 / 26.29	15.58 / 15.53	191.6 / 191.3	1.64	1.97	1.88–2.04

Table S7. Admissibility analysis for the group means of Table S1 (model-based). The admissible W_{14} range is obtained by linear programming over all curves consistent with F_{max}, $u(F_{max})$, and k (assumed 2–8 mm window); the force range at 6 mm is obtained with W_{14} additionally fixed.

Variant	Measured mean W_{14} [N mm]	Admissible W_{14} for given F_{max}, $u(F_{max})$, k [N mm]	Admissible range [% of W_{14}]	Force range at 6 mm with W_{14} also fixed [N]
PLA	577.5	434–766	57	30.7–44.1
Impact PLA	590.8	424–714	49	35.3–49.2
PETG	622.8	306–681	60	41.2–52.4
ABS	451.5	227–546	71	27.0–41.8
ASA	449.6	157–529	83	27.7–40.8
UltraT	572.1	412–683	47	34.6–45.3
PA12 (FDM)	355.5	295–598	85	16.8–26.0
PA12 (SLS)	433.3	338–675	78	17.6–31.1
Flex 3D	187.2	116–321	110	5.9–13.9

Table S8. Global effective Poisson's ratio ν_{eff} of the idealized linear finite-element model described in Section 2.6 [37] (30 N; normalized by W_0 = 80 mm and L_0 = 176.88 mm). These are model results; the PLA material models are specified in Section 2.6 (isotropic: E = 2939 MPa, ν = 0.33; orthotropic constants as listed there), and the values are presented separately from the optical observations in Table 3.

Material model	FEM ν_{eff} [–]
PLA (isotropic)	−1.032
PLA (orthotropic)	−1.033
Impact PLA	−1.032
PETG	−1.031
ABS	−1.035
ASA	−1.031
UltraT	−1.031
PA12 (FDM)	−1.031
Flex 3D	−1.032

Reference

[37] Schürger, B. Design and Implementation of Metamaterials in Relation to Their Mechanical Properties. Ph.D. Thesis, Technical University of Košice, Faculty of Mechanical Engineering, Košice, Slovakia, 2026.
